# Daily Associations of Air Pollution and Pediatric Asthma Risk Using the Biomedical REAI-Time Health Evaluation (BREATHE) Kit

**DOI:** 10.3390/ijerph19063578

**Published:** 2022-03-17

**Authors:** Hua Hao, Sandrah P. Eckel, Anahita Hosseini, Eleanne D. S. Van Vliet, Eldin Dzubur, Genevieve Dunton, Shih Ying Chang, Kenneth Craig, Rose Rocchio, Theresa Bastain, Frank Gilliland, Sande Okelo, Mindy K. Ross, Majid Sarrafzadeh, Alex A. T. Bui, Rima Habre

**Affiliations:** 1 Department of Population and Public Health Sciences, University of Southern California, Los Angeles, CA 90039, USA; hhao@usc.edu (H.H.); eckel@usc.edu (S.P.E.); eldind@gmail.com (E.D.); dunton@usc.edu (G.D.); bastain@usc.edu (T.B.); gillilan@usc.edu (F.G.); 2 Department of Computer Science, University of California Los Angeles, Los Angeles, CA 90095, USA; ani.dachia@gmail.com (A.H.); majid@cs.ucla.edu (M.S.); 3 Health Effects Institute, Boston, MA 02110, USA; evanvliet@healtheffects.org; 4 Sonoma Technology, Inc., Petaluma, CA 94954, USA; cchang@sonomatech.com (S.Y.C.); kcraig@sonomatech.com (K.C.); 5 Mobilize Labs, University of California Los Angeles, Los Angeles, CA 90095, USA; rrocchio@oarc.ucla.edu; 6 Department of Pediatrics, University of California Los Angeles, Los Angeles, CA 90095, USA; sokelo@mednet.ucla.edu (S.O.); mross@mednet.ucla.edu (M.K.R.); 7 Medical & Imaging Informatics Group, Department of Radiological Sciences, University of California Los Angeles, Los Angeles, CA 90095, USA; buia@mii.ucla.edu

**Keywords:** personal air pollution, pediatric asthma, sensors, GPS, mobile health, PRISMS

## Abstract

Background: Exposure to air pollution is associated with acute pediatric asthma exacerbations, including reduced lung function, rescue medication usage, and increased symptoms; however, most studies are limited in investigating longitudinal changes in these acute effects. This study aims to investigate the effects of daily air pollution exposure on acute pediatric asthma exacerbation risk using a repeated-measures design. Methods: We conducted a panel study of 40 children aged 8–16 years with moderate-to-severe asthma. We deployed the Biomedical REAI-Time Health Evaluation (BREATHE) Kit developed in the Los Angeles PRISMS Center to continuously monitor personal exposure to particulate matter of aerodynamic diameter < 2.5 µm (PM_2.5_), relative humidity and temperature, geolocation (GPS), and asthma outcomes including lung function, medication use, and symptoms for 14 days. Hourly ambient (PM_2.5_, nitrogen dioxide (NO_2_), ozone (O_3_)) and traffic-related (nitrogen oxides (NO_x_) and PM_2.5_) air pollution exposures were modeled based on location. We used mixed-effects models to examine the association of same day and lagged (up to 2 days) exposures with daily changes in % predicted forced expiratory volume in 1 s (FEV_1_) and % predicted peak expiratory flow (PEF), count of rescue inhaler puffs, and symptoms. Results: Participants were on average 12.0 years old (range: 8.4–16.8) with mean (SD) morning %predicted FEV_1_ of 67.9% (17.3%) and PEF of 69.1% (18.4%) and 1.4 (3.5) puffs per day of rescue inhaler use. Participants reported chest tightness, wheeze, trouble breathing, and cough symptoms on 36.4%, 17.5%, 32.3%, and 42.9%, respectively (*n* = 217 person-days). One SD increase in previous day O_3_ exposure was associated with reduced morning (beta [95% CI]: −4.11 [−6.86, −1.36]), evening (−2.65 [−5.19, −0.10]) and daily average %predicted FEV_1_ (−3.45 [−6.42, −0.47]). Daily (lag 0) exposure to traffic-related PM_2.5_ exposure was associated with reduced morning %predicted PEF (−3.97 [−7.69, −0.26]) and greater odds of “feeling scared of trouble breathing” symptom (odds ratio [95% CI]: 1.83 [1.03, 3.24]). Exposure to ambient O_3_, NO_x_, and NO was significantly associated with increased rescue inhaler use (rate ratio [95% CI]: O_3_ 1.52 [1.02, 2.27], NO_x_ 1.61 [1.23, 2.11], NO 1.80 [1.37, 2.35]). Conclusions: We found significant associations of air pollution exposure with lung function, rescue inhaler use, and “feeling scared of trouble breathing.” Our study demonstrates the potential of informatics and wearable sensor technologies at collecting highly resolved, contextual, and personal exposure data for understanding acute pediatric asthma triggers.

## 1. Introduction

Asthma affects more than 25 million Americans, representing 8% of adults and 7% of children [1]. Asthma prevalence has been increasing over the last few decades in all age, sex, and racial groups, especially in children [2]. Children with asthma are significantly burdened by asthma morbidity, with higher rates of emergency department visits, hospitalizations, and deaths [3,4]. A large proportion of the asthma burden is the consequence of poor asthma control [5]. Children with poorly controlled asthma report a decreased health-related quality of life [6]. Asthma guidelines emphasize the importance of achieving asthma control to minimize or prevent exacerbations; however, several asthma triggers are known to exacerbate asthma resulting in increased symptoms, reduced lung function, and the need to use rescue inhaler medications [7,8]. Major determinants of the severity and persistence of asthma described in the literature include genetics [9], atopy [10], pollution [11], environmental tobacco smoke [12], respiratory infections [13], etc.

Environmental exposures, including air pollution and reduced temperature or humidity, are among the many recognized asthma triggers [14]. Previous studies exposure to ozone (O_3_), nitrogen dioxide (NO_2_), and particulate matter (PM) may induce or aggravate asthma [15], and air pollution exposure is associated with increased emergency department visits for asthma [16], reduced %predicted forced expiratory volume in 1 s (FEV_1_) [17], rescue medication use [18], and increased cough and wheeze symptoms [19].

Evidence on within-person acute effects (daily to sub-daily) of air pollution exposure on asthma is more limited than evidence on chronic effects, largely because of the challenges involved in collecting highly resolved exposure and outcome information over extended periods of time at a personal level. However, advances in mobile health (mHealth) technologies including wearables, sensors, smartphone applications (apps), and informatics are enabling these studies. Toward this goal, the Biomedical REAL-Time Health Evaluation (BREATHE) Kit was developed in the Los Angeles Pediatric Research Using Integrated Sensor Monitoring Systems (PRISMS) Center as a sensor-based informatics platform for environmental health studies of pediatric asthma to enable such studies [20]. In this first analysis, we deployed the BREATHE Kit in a panel study aimed to investigate the association of acute (daily) air pollution exposure with risks of reduced lung function, rescue inhaler use, and increased symptoms in children with moderate-to-severe asthma [20]. Subsequent research will expand these analyses to investigate the association of sub-daily, acute, and peak exposures on pediatric asthma risk.

## 2. Materials and Methods

### 2.1. Study Design and Population

**Figure 1:**
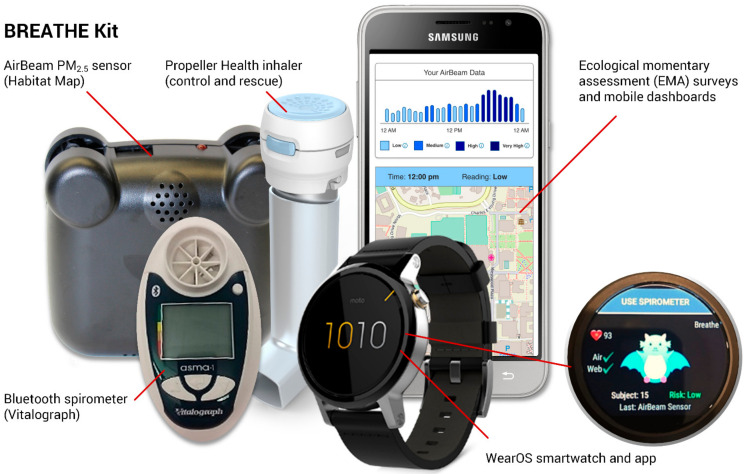
The Biomedical REAL-Time Health Evaluation (BREATHE) Kit developed in the Los Angeles PRISMS Center.

We recruited 40 children with moderate-to-severe asthma from the University of California, Los Angeles Pediatric Asthma Center of Excellence clinics located in Los Angeles, CA, and Santa Monica, CA, from February 2019 through December 2019. Eligibility criteria included English-speaking children aged 8–16 years with doctor-diagnosed asthma. Participants were prescreened for eligibility based on their medical records and recruited by a dedicated study coordinator within the clinic during their routine appointments. Each child was given a Biomedical REAL-Time Health Evaluation (BREATHE) Kit [20] (Figure 1), which included an Android smartphone (Samsung S4) with a custom app to display sensor data and deliver Ecological Momentary Assessment (EMA) surveys; a smartwatch (Motorola Moto 360) with a custom app; an Airbeam I (HabitatMap) personal air pollution exposure sensor (measuring PM_2.5_ particulate matter less than 2.5 µm in aerodynamic diameter, relative humidity, and temperature); handheld spirometer (Asma-1 BT, Vitalograph Inc., Lenexa, KS, USA); rescue and control medication inhaler sensors (Propeller Health, Inc., Madison, WI, USA) matched to their medication regimen. Every data point collected with the BREATHE Kit is geotagged with latitude and longitude coordinates and location metadata and timestamped, as described in more detail in Bui et al. [20].

Children and their caretakers were trained on how to properly use and charge the BREATHE Kit and its components in the clinic, including how to initiate and perform proper spirometry maneuvers, respond to smartphone surveys, charge devices, and verify data communications connectivity. The day following recruitment, a detailed baseline questionnaire was conducted over the phone with the child and their caregiver to collect asthma-related health and environmental data (e.g., typical activity patterns of the child, household ventilation conditions, indoor sources of air pollution, etc.).

Subjects were monitored for 14 days during which data collection and transmission status were continuously monitored in real time by the research coordinator in a dedicated researcher dashboard. When the researcher dashboard indicated sensors were offline or missing data for an extended period of time (generally 1+ days), participants were contacted to help troubleshoot issues or encourage compliance with data collection. Once the monitoring period was completed, participants mailed their kits back in prelabeled packages and completed an interviewer-administered exit survey over the phone asking about their experience with the BREATHE Kit and the study. The institutional review board of the University of California Los Angeles approved the study protocol (Protocol #15-001402). Informed consent and assent were obtained in the clinic from the primary legal guardian accompanying the child and the child participant, respectively, upon recruitment.

### 2.2. Asthma Outcomes

#### 2.2.1. Lung Function

Subjects measured their forced expiratory volume in 1 s (FEV_1_) and peak expiratory flow (PEF) twice a day at home using the Vitalograph Asma-1 BT Bluetooth-enabled monitor. The handheld device provided immediate feedback (beeping tone and visual symbol on display) to indicate when a “good” maneuver was obtained. These data on maneuver quality were captured with the lung readings and transmitted to the BREATHE Kit in real time. Participants were instructed to collect three “good” maneuvers in the morning and in the evening every day, with up to six attempts per session, at the same time every day. Morning and evening measurement times were decided upon in the clinic during recruitment based on typical wake and sleep times, within predefined time windows.

The maximum (best effort) lung function was calculated from at least two reproducible maneuvers (as the average of the two) and selected for analysis. If no reproducible value was found, the maximum value was selected. We calculated % predicted lung function based on age, sex, and height for FEV_1_ and PEF based on equations from Knudson et al. [21].

We also fit a linear regression of FEV_1_ measured using clinical-grade spirometers (Morgan rolling seal LT spirometers, Morgan Scientific, Inc., Haverhill, MA, USA) at the clinic during the recruitment appointment (or closest date retrieved from medical records when not scheduled for the same day) on FEV_1_ measured after coaching with the Asma-1 BT in the participant’s BREATHE Kit to assess the correlation between the two as an indicator of data quality.

#### 2.2.2. Inhaler Use

We provided Bluetooth^®^-enabled Propeller Health Inc. inhaler sensors integrated with the BREATHE Kit to track puffs of controller and rescue inhaler dispensed. Sensors were attached to the participant’s medications, tested for data connectivity, and demonstrated in the clinic upon recruitment. We aimed to provide one sensor per regularly used inhaler medication; however, compatible sensors were not always available for every type of medication. If participants did not bring their medications to the clinic visit, their parent/caretaker was provided with a sensor(s) and instructed to attach and test it at home with phone coaching by the study coordinator. However, this self-setup scenario was more technically challenging for participants and did not always result in properly connected sensors. Medication data were summarized on a person-day level for rescue (puffs count, modeled as outcome) and control inhaler use (binary for any use, adjusted for as potential confounder in health models described below).

#### 2.2.3. Asthma Symptoms

Self-reported asthma symptoms were collected via ecological momentary assessment (EMA) surveys deployed on a custom app with the BREATHE Kit using four types of surveys (morning/end of day, after-school (on weekdays), random (within predesignated 2 h windows), and context-sensitive sensor-triggered) to minimize recall bias, maximize validity, and capture participants’ symptoms in context. A detailed survey prioritization and suppression logic was designed to manage the burden on participants while capturing potentially rare or important events [20]. Briefly, scheduled (morning/after-school/end of day) and inhaler- or lung-function-sensor-triggered surveys were prioritized. A limit of ≤1 per 10 min (inhaler) and ≤1/h (lung function) were imposed. Random and remaining context-sensitive surveys (detailed below) were managed with a variety of limits, including a daily cap (maximum 2/day), ≤1/h, 10 min rest period in between any consecutive survey, and a 2 h density limit. The following questions based on the Asthma Control Test (ACT) [22] were asked but rephrased to refer to either the past hour, the previous night, the day, or during school time.

Morning surveys: Questions on asthma experienced in the previous night were delivered at respondents’ wake-up time as determined by participants and their caretaker at recruitment (between 6.30 a.m. and 9.00 a.m.). These included the following: “Did you wake up because of your asthma?” (response choices: Yes/No); “How many times did you use your inhaler during the night?” (Never/One time/Two times/Three times/Four or more times, which was recorded to Never/One or more times in this analysis).

Random and sensor-triggered surveys: Additional asthma symptoms were collected via EMAs surveys sent out at random times within predesignated 2 h windows throughout the day or triggered in real time based on sensor data streams. Context-sensitive surveys were triggered after rescue inhaler use, lung function testing, peak in PM_2.5_ concentrations, and following a sustained (>5 min) increase in heart rate corresponding to moderate exercise intensity (defined as heart rate (average in 2 min sliding window) > 0.5 (HR_max_ − HR_rest_) + HR_rest_, where HR_rest_ is calculated based on child’s age and resting heart rate collected at recruitment. These are described in more detail in Bui et al. [20]. Questions on asthma symptoms in the last hour included the following: “In the past hour, did your chest feel tight because of asthma?”; “In the past hour, did you feel wheezy (whistling in the chest) because of asthma?”; “In the past hour, did you have trouble breathing because of your asthma?”; “In the past hour, did you cough because of your asthma?”; “In the past hour, how much of a problem was your asthma when you ran, exercised or played sports?”; “In the past hour, did you feel scared that you might have trouble breathing because of your asthma?”; and “In the past hour, have you avoided strenuous activities, or had to slow down or stop exercising because of your asthma?” Response choices for all these questions were “Not at all/A little/Quite a bit/Very much so” except for asthma being a problem when exercising which included an additional option of “I did not run, exercise, or play sports.” Dichotomous symptom variables were created at the daily level as “Not at all” in all completed surveys versus any other report (A little, Quite a bit, Very much so).

After School: Questions were the same as the random survey described above, except they started with “At school today” instead of “In the past hour.”

End of the day: This last EMA survey of the day was scheduled for 7:00 p.m. and collected day-level information. Questions included the random survey symptom questions (referring to the past hour) as well as the following: “How much of the time did your asthma keep you from getting as much done at school or at home today?” Response choices were summarized similarly to the random survey questions at the daily level.

### 2.3. Environmental Exposure Assessment

Personal monitoring. Personal exposure to particulate matter with aerodynamic diameter < 2.5 µm (PM_2.5_), relative humidity (RH), and temperature were continuously measured using the AirBeam 1.0 (HabitatMap) following the BREATHE Kit energy optimization cycle (15 secs data collection every 1 min) [20]. A running median filter (within a centered window of 10 observations) was applied to personal RH to remove outliers. As the degree of missingness in personal RH was high, we developed a model to impute it on the person-day level for use in health models. The imputation used the following predictors in a mixed-effects model: daily ambient RH (calculated from ambient dew point and temperature [23], described below), daily ambient temperature, and the visit-level difference between mean ambient and personal RH. The model included a random intercept for a subject to account for person-level clustering in the data. It also included a random slope for ambient RH to allow the relationship between daily ambient and personal RH to vary by person depending on their typical activity patterns or household characteristics. The Pearson correlation between daily predicted and measured personal RH was 0.97.

Modeled ambient air pollution and meteorology. An Environmental Data Web Service was built by Sonoma Technology Inc. (STI, Petaluma, CA, USA), to provide real-time and archived weather (ambient temperature and dew point) and ambient air pollution data streams based on user location (determined by GPS), date, and hour to support the BREATHE Kit. Meteorological data were extracted from the NOAA Real-Time Mesoscale Analysis (RTMA) hourly, 2.5 km × 2.5 km data assimilation product (https://www.nco.ncep.noaa.gov/pmb/products/rtma/#RTMA2p5, accessed 5 June 2020). Hourly ambient air pollution exposures were modeled using inverse-distance squared spatial interpolation from surrounding regulatory monitors for PM_2.5_, ozone (O_3_), nitrogen dioxide (NO_2_), nitrogen oxide (NO), and nitrogen oxides (NO_x_). These reflect hourly concentrations of air pollutants from general background or regional sources. Los Angeles, CA, has one of the densest regulatory ambient air monitoring networks in the US, which provided comprehensive coverage in our study area.

Modeled traffic-related air pollution. Air pollutant concentrations from vehicular traffic on nearby roads, referred to as traffic-related PM_2.5_, NO_x_, and NO_2_, were modeled using the RLINE line source dispersion model and provided in the STI web service. RLINE uses local weather data and a comprehensive database of roadways, annual traffic volume, and vehicle emission factors for southern California to estimate pollutant concentrations contributed by on-road mobile source emissions at the participant receptor points [24].

Daily exposure averaging windows. To investigate the association of these exposures with morning, evening, and day-average outcomes, two averaging intervals were used to calculate 24 h exposure averages that precede the outcomes as follows: For morning outcomes such as morning lung function, 24 h averages were calculated starting from 6.00 a.m. the previous day to 6.00 a.m. of the current day. For evening (e.g., evening lung function) and daily outcomes (e.g., daily average lung function), 24 h averages were calculated starting from 6.00 p.m. the previous day to 6.00 p.m. of the current day. The cut points of 6.00 a.m. and 6.00 p.m. were selected because most participants completed their morning and evening lung function tests after 6.00 a.m. and 6.00 p.m., respectively (Supplementary Figure S1). Moreover, 24 h averages were calculated using 30% completeness criteria, which is more relaxed than typical air pollution investigations utilizing modeled ambient data given the greater chance of missing data using personal sensors and real-time data transmission.

### 2.4. Covariate Information

Based on previous air pollution and asthma literature [9,10,11,12,13], we considered the following covariates a priori as potential confounders: sex, race, Hispanic ethnicity, caretaker’s education level, household income, personal and ambient relative humidity and temperature, subject’s person-day level time–activity patterns, asthma medication use, outdoor physical activities, exposure to smoking (exposure to secondhand smoking in the home and in utero exposure to maternal smoking), home characteristics (kitchen ventilation, fuel use, presence of pets) and day of the week. Of this list, only Hispanic ethnicity and personal relative humidity were selected to be included in the final model since they were significant predictors, and their inclusion resulted in greater than roughly 10% change in the main pollutant effect estimate. This decision was guided by a priori selection of potential covariates and balanced the need to minimize degrees of freedom in the models and ensure comparable adjustments across health models. Given the importance of asthma control on the risk of these outcomes, we further adjusted for same-day use of controller medication in sensitivity analyses as a potential confounder.

### 2.5. Statistical Analysis

Spearman correlations were calculated to assess correlations between different pollutants given their non-normal distribution. We tested the association between daily air pollution exposures and lung function (% predicted FEV_1_ and PEF), the daily count of rescue inhaler medication use, and asthma symptoms using mixed-effects models with a random intercept for each subject to account for the repeated-measures design. We investigated these associations for the preceding 24 h (lag 0) as well as lags 1 and 2 days. Daily lags followed the 6.00 a.m. to 6.00 a.m. or 6.00 p.m. to 6.00 p.m. definitions explained above, depending on whether the outcome was assessed in the morning or during the day/in the evening, respectively. For example, lag 1 was defined as the preceding 25–48 h and lag 2 as the preceding 49–72 h for daily or evening outcomes.

We reported results as a change in %predicted value for lung function, the rate ratio for rescue inhaler use, and odds ratios of experiencing symptoms. All effects estimates were scaled to a standard deviation (SD) increase in each pollutant (based on lag 0 distributions) to allow standardized inter-pollutant comparisons of health effects. We also fit two-pollutant models to test whether pollutant effects were potentially confounded by co-exposure to another pollutant, in cases in which the two pollutants were not highly correlated (Spearman correlation < 0.5). Statistical significance was determined based on a *p*-value < 0.05. All analyses were conducted in SAS 9.3 (SAS Institute Inc., Cary, NC, USA).

## 3. Results 

### 3.1. Descriptive Summaries

Children were 12 years old on average (range 8–16 years, *n* = 40), 45% female, and 42.5% of Hispanic ethnicity. Descriptive statistics are presented in Table 1 and Supplementary Table S1. All participants were enrolled for 14 days in the study, except for 3 who withdrew and only completed 2, 3, and 5 days of follow-up, respectively. Overall, 1172 spirometry maneuvers were attempted and 887 (76%) were classified as “good”. Of 218 person-days with lung function data, 78 (36%) achieved a minimum of 6 attempts (3 maneuvers in each of morning and evening test sessions as instructed). Among days with <6 attempts, a median of three “good” attempts was obtained. On average, %predicted FEV_1_ and PEF were lower in the morning and increased in the evening (Table 2). The fitted regression line between the FEV_1_ measurements obtained with the Asma-1 BT handheld spirometer on recruitment day and clinic measurements using clinical-grade spirometers had an R^2^ = 0.68 (Supplementary Figure S1). On average, subjects used 1.4 puffs/day of rescue medication, with a range of 0–24 puffs/day (Table 2 and Supplementary Figure S2). In general, subjects answered more morning and end-of-day symptom survey questions compared to random surveys (Table 2).

Measured personal exposures had greater missingness than modeled ambient and traffic-related environmental exposures (Supplementary Table S2). Concentrations of personal 24 h PM_2.5_ were highly variable between and within subjects, with a maximum reaching 64.7 µg/m^3^ (Supplementary Figure S3). Personal PM_2.5_ exposure was also more variable than ambient PM_2.5_. Personal and ambient PM_2.5_ were moderately correlated (Spearman r = 0.39), while personal and traffic-related PM_2.5_ were weakly correlated (r = 0.14). Ambient pollutants had moderate-to-high correlations with each other, similar to traffic-related pollutants. However, ambient O_3_ was weakly correlated with remaining pollutants (Supplementary Table S3).

### 3.2. Air Pollution and Health Models

**Figure 2:**
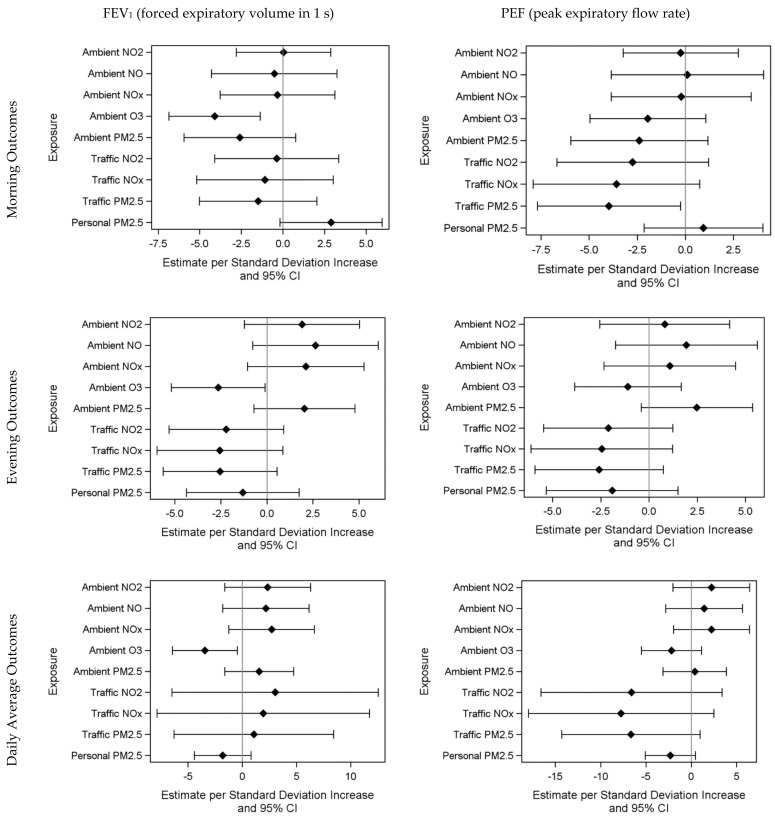
Association of daily air pollution exposure (lag 0) with %predicted FEV_1_ and PEF. Effect estimates and 95% confidence intervals are scaled to a standard deviation change in exposure. Effect estimates were scaled to a standard deviation change in pollutant concentrations as follows: personal PM_2.5_: 9.1 μg/m^3^; traffic-related PM_2.5_: 0.7 μg/m^3^; traffic-related NO_x_: 14.7 ppb; traffic-related NO_2_: 7.1 ppb; ambient PM_2.5_: 3.7 μg/m^3^; ambient O_3_: 9.2 ppb; ambient NO_x_: 6.1 ppb; ambient NO: 2.4 ppb; ambient NO_2_: 4.6 ppb.

Final sample sizes for different outcome models varied based on exposure and outcome data completeness from *n* = 39 to 167 person-days (in the ambient O_3_ (lag 0) and rescue inhaler use model). Supplementary Table S4 presents results of %predicted FEV_1_ models (interpreted as a percentage point change). One SD increase in same-day (lag 0) O_3_ (9.2 ppb) was associated with a 4.11% lower [95% CI: −6.86, −1.36] morning %predicted FEV_1_. O_3_ was also significantly inversely associated with evening (−2.65 [−5.19, −0.10]) and daily average %predicted FEV_1_ (−3.45 [−6.42, −0.47]). Similarly, lag 1 (previous day) O_3_ exposure was associated with lower evening (−4.90 [−7.94, −1.85]) and daily %predicted FEV_1_ (−4.92 [−8.44, −1.40]); however, only the association with morning %predicted FEV_1_ marginally remained at lag 2 (−2.94 [−5.93, 0.05]). For traffic-related pollutants (PM_2.5_, NO_x_, NO_2_), lag 1 exposure was significantly and inversely associated with morning %predicted FEV_1_. Overall, most same-day (lag 0) air pollutant exposures were inversely associated with morning %predicted FEV_1_, although some were not significant (Figure 2).

Supplementary Table S5 presents results for %predicted PEF. Previous-day (lag 1) traffic-related PM_2.5_ exposure was associated with 3.97% lower [95% CI: −7.69, −0.26] morning %predicted PEF per SD (0.7 µg/m^3^). Associations were marginal at lag 1 (−3.35 [−6.89, 0.19]) and lag 2 (−6.27 [−12.75, 0.21]). Traffic-related NO_x_ and NO_2_ were significantly associated with lower morning PEF at lag 1 (traffic-related NO_x_: −4.91 [−9.28, −0.54], traffic-related NO_2_: −4.57 [−8.51, −0.63]). Personal PM_2.5_ exposure was not significantly associated with lung function (Figure 2). Adjusting for control inhaler use did not meaningfully change any of the observed results (Supplementary Tables S6 and S7).

Supplementary Table S8 reports associations between rescue inhaler use and air pollutants. Most significant associations were found for same-day (lag 0) exposures. Ambient air pollutants (PM_2.5_, O_3_, NO_x_, NO, NO_2_) on lag 0 days were all positively associated with daily rescue inhaler use; however, this association was not significant for ambient PM_2.5_ and NO_2_. An increase of 1 SD (9.2 ppb) in O_3_ was associated with 1.52 times greater rate of rescue inhaler use [95% CI: 1.02, 2.27]. In contrast, same-day exposure to traffic-related PM_2.5_, NO_x_, NO_2_ was significantly negatively associated with rescue inhaler use. However, these associations became non-significant in two-pollutant models adjusted for O_3_ (Supplementary Figure S4). Although positive, personal PM_2.5_ on lag 0 was not significantly associated with rescue inhaler use.

Supplementary Table S9 presents findings for asthma symptoms. One-SD increase in traffic-related PM_2.5_ (lag 0) was significantly associated with 83% (95% CI: 3%, 224%) higher odds of feeling scared of having trouble breathing because of asthma. Most associations were variable and not significant for cough, wheeze, chest tightness, trouble breathing, avoiding strenuous activities because of asthma, and asthma interfering with daily activities, although sample sizes for symptoms were more limited than the other outcomes (ranged from 52 person-days for personal PM_2.5_ models to maximum 154 person-days for other pollutants). Finally, Supplementary Table S10 presents results for all outcomes in models adjusted for parental asthma status, caretaker education level, Hispanic ethnicity, and personal relative humidity to compare to the final models presented in this analysis.

## 4. Discussion

Our findings revealed significant associations between same-day and previous-day exposure to air pollution and risk of reduced lung function, rescue inhaler use, and symptoms in a panel study of 40 children with moderate to severe asthma in Los Angeles, CA. In general, we found the strongest associations for O_3_ and traffic-related PM_2.5_ with lung function, and with ambient pollutants and rescue inhaler use. Although limited in sample size, the risk of feeling scared of having trouble breathing because of asthma was also associated with traffic-related PM_2.5_ exposure. In contrast, total personal PM_2.5_ exposure—although marginal in some cases—was not associated with any of our outcomes. To the best of our knowledge, our study is the first to deploy a wearable, sensor-based informatics platform to monitor and model such an extensive suite of environmental exposures and potential acute asthma triggers at the personal level, in context, and with such high spatiotemporal resolution. As such, important data considerations, methodological challenges, and lessons learned will also be shared and discussed below.

For lung function, our findings generally agree with the literature, although the effects of ambient versus traffic-related pollutants were more pronounced for FEV_1_, while the inverse was true for PEF. Several studies reported short-term exposure to O_3_ was associated with lung function decrements [25,26,27,28], which is consistent with our O_3_ and FEV_1_ results. Ozone is a strong oxidant that is formed in the troposphere via chemical reactions in the presence of precursor pollutants, such as volatile organic compounds, nitrogen oxides, and solar radiation [29]. Ozone concentrations are generally higher outdoors compared with indoors; therefore, human exposures to ozone mainly occur in the outdoor environment [30]. The World Health Organization (WHO) [31] states that there is more consistent evidence on the short-term rather than the long-term effects of O_3_, which include increases in daily mortality and morbidity, especially for respiratory causes [32]. As up to 90% of inhaled ozone is absorbed in the respiratory tract along the bronchial tree [33], O_3_ responses are likely initiated and localized in the respiratory tract lining fluid due to the low solubility and high reactivity of O_3_ [34]. Once inhaled, O_3_ reacts with proteins and lipids of the lung lining fluid resulting in cytokines generation leading to an increase in lung permeability and edema development [35]. Consequently, O_3_ exposure is believed to result in acute oxidative stress and lung inflammation, contributing to respiratory morbidities such as reduced lung function [36]. The range of magnitude in the association we found between short-term O_3_ and evening FEV_1_ is comparable to previous studies, with a lower limit of −5.2% expected reduction in %predicted FEV_1_ (−2.65 [−5.19, −0.10]). For example, a previous literature review reported that short-term O_3_ exposure was associated with a wide range of reduction in %predicted FEV_1_ in children (−0.01% to −9% reduction per 10 ppb change in O_3_), for periods ranging from 1 day to 2 weeks [37]. Similarly, expected changes as large as −9.3% reduction in morning %predicted FEV_1_ and −9.3% reduction in morning %predicted PEF were seen with lag 1 traffic-related NO_x_ exposure (lower 95% CI limit, Supplementary Tables S4 and S5). Similarly, rate ratios of rescue inhaler use were as high as 2.3 for same-day ambient O_3_ exposure and 2.4 for same-day ambient NO exposure (upper 95% CI, Supplementary Table S8). The full confidence interval should, therefore, be taken into consideration in the interpretation of our results, which could range from subtle to more noticeable effects on morbidity and quality of life for children with asthma on a day-to-day basis.

A 2001 study in The Netherlands reported significant decreases in PEF in children following exposures to PM_10_ (lags 1 and 3 days), NO_2_ (lags 0 and 1), and NO (lag 3) [38]. In our study, we found same-day and up to 2 days lag exposure to traffic-related pollutants (PM_2.5_ on lag 0, 1, 2 days; NO_x_ and NO_2_ on lag 1 day) was associated with reduced morning PEF. Traffic is one of the most important sources of NO_x_ in southern California, and a large contributor to overall PM_2.5_ concentrations [38]. Although other pollutants could be present in the near-roadway mixture and could contribute to these effects, in general, our findings seem consistent with the literature. Moreover, Li et al. [39] reviewed more than 30 panel studies on the effects of air pollution on children’s lung function and respiratory symptoms. They reported that PM and NO_2_ showed more significant associations with PEF, but findings for many outcomes depended on the number of lag days similarly to our results.

In addition, previous studies investigated the biological mechanisms contributing to decreased lung function as a result of PM_2.5_ exposure. They reported that PM_2.5_ could reach the alveoli, and up to 50% may remain in the lung tissue [40]. Additionally, because of their deep deposition in the alveoli, their removal or clearance rate (via mucociliary transport) can be slow [41]. This further supports lagged effects and could provide actionable information to physicians to consider warning pediatric asthma patients and parents to be more vigilant about symptom monitoring both on particular poor air quality days, as well as for the following 1–2 days, to adequately prepare for potential symptom flares.

Similarly, several previous studies reported daily associations of ambient PM, O_3_, and NO_2_ exposure with rescue inhaler use [42,43], consistent with our findings for O_3_, NO_x_, and NO. However, ambient PM_2.5_ was positively but non-significantly associated with rescue inhaler use in our study. Existing evidence suggests that short-term exposure to O_3_ and NO_2_ can cause airway inflammation, reduced pulmonary function, and exacerbation in individuals with asthma [44]. While consistent with the literature, it is possible our study was underpowered to detect significant associations with ambient PM_2.5_. Exposure measurement error inherent in the ambient and traffic-related estimates could also lead to weaker statistical power to detect effects in our study. Ambient PM_2.5_ does not fully capture personal exposure to PM_2.5_ of outdoor origin. Ambient and personal PM_2.5_ were weakly correlated in our data (r = 0.39), which also suggests personal PM_2.5_ exposures in our population are heavily impacted by indoor and personal activity-related sources and do not correlate with outdoor variations in ambient PM. For traffic-related PM_2.5_, NO and NO_x_, we found increased exposure to those pollutants was associated with a decreased rate of rescue inhaler use. However, after we adjusted ozone in the same model, these associations became non-significant, but the point estimate was still negative. The possible explanation could be residual confounding due to other co-occurring exposures, behaviors, or time–activity patterns.

As for symptoms, sample sizes available for analysis were generally much lower relative to other sensor-measured outcomes, likely due to the additional burden involved in actively responding to multiple EMA surveys during the day. We only found significant associations between exposure to traffic-related PM_2.5_ and feeling scared of having trouble breathing. Despite a roughly similar sample size, we did not find significant associations of traffic-related PM_2.5_ with daily trouble breathing symptoms. One possible explanation could be the increased perception of risk or anticipation of an asthma attack when children are exposed to traffic for long periods of time, perhaps based on prior experiences, and thus might increase stress and anxiety levels. Several studies reported the associations between increased air pollution levels and asthma symptoms, and limited research has been conducted on the symptom of trouble/difficulty breathing. One study in Spokane, WA, investigated exposure to PM in several sizes and several asthma symptoms in children, including trouble breathing [45]. They found positive but non-significant results, similar to our study. We also found positive but non-significant associations between ambient PM_2.5_ and some traffic-related air pollutants with cough, wheeze, asthma being a problem when running, exercising, or playing sports. Cough is a commonly studied asthma symptom in many previous studies [46,47,48], but with inconsistent findings. One study in New York found stronger daily associations of PM of indoor origin with wheeze, while O_3_ and PM of outdoor origin were more strongly associated with cough [19]. However, given the limited sample size for symptoms and personal PM_2.5_ in our study, we were not able to further model or disentangle the effects of PM_2.5_ by origin or source. The higher concentrations and variability of personal PM_2.5_, compared with ambient, further illustrate the diverse and complex sources, behaviors, and factors that contribute to true personal exposure which are often missed by using ambient estimates. Since PM composition and toxicity can vary considerably based on its contributing sources, this is an important future direction of research.

As for exposure patterns, ambient pollutants were more highly correlated with personal PM_2.5_ exposure than traffic-related pollutants. This could be due to participants’ time–activity patterns and home operation characteristics (e.g., window opening), leading to infiltration of outdoor air pollution indoors. It is also possible that study participants did not spend too much time in transit or commuting during the monitoring period, or when they did, they were more isolated or protected from traffic emissions by operating air conditioners and closing car windows. We did not have data to ascertain these behaviors but believe this is possible for a pediatric population with moderate-to-severe asthma living in Los Angeles, CA.

Our study includes a number of limitations. First, the personal PM_2.5_ exposure was measured using commercial grade sensors that were challenging or not feasible to regularly calibrate while the study was operational. Low-cost optical PM sensors such as the one used in this study tend to overestimate PM_2.5_ concentrations largely due to relative humidity interferences. In addition, they do not capture particles <~300 nm in aerodynamic diameter, and their response can vary based on the actual chemical composition and size distribution of the aerosol mixture [49]. Whether or not the overestimation can have any effect on the health model findings could be assessed in future analyses; however, it is unlikely to be differential in relation to the outcome. Second, although we relied on real-time data streaming checks built into the researcher dashboard to regularly manage and encourage compliance with data collection, on top of the built-in engagement features of the BREATHE Kit, it is still possible that participants were not always fully compliant in using or charging their devices or not always allowed to take them to school. These challenges—while common in personal monitoring studies—require more research in terms of encouraging and rewarding compliance and designing mHealth platforms with users in mind to balance burden with quality, completeness, and representativeness of data. We also recommend careful evaluation of temporal and spatial patterns of data missingness in future mHealth studies to determine whether these might correlate with behaviors or outcomes being assessed and might introduce bias. For example, device use or battery power might correlate with certain times of day, for example, or certain microenvironments (e.g., home) might be more represented in the data.

In addition, the generalizability of our findings is potentially limited. Our participants were recruited from a pediatric asthma specialty clinic and had moderate-to-severe asthma. As such, triggers and factors associated with exacerbations in this population might not necessarily translate to children with mild asthma or well-controlled asthma. In addition, the socioeconomic characteristics of our study sample are likely not representative of the general population or more disadvantaged or environmentally burdened populations, as 37.5% of participants’ caretakers had graduate-level degrees, 57.5% of participants’ caretakers reported over USD 50,000 household income annually, and all participants had health insurance coverage. Finally, selection bias may have influenced our results if parents living in areas with higher air pollution exposure were also more concerned about its impact on their child’s asthma and therefore more likely to participate in our study. However, it is difficult to ascertain the direction of this potential selection bias.

Strengths of our study include a repeated-measures design that allowed us to investigate associations at the daily, within-person level over an extended period of 14 days, in a highly susceptible pediatric asthma population. This is also the first study up to our knowledge to deploy a sophisticated sensor-based informatics platform that allowed us to continuously collect very highly spatiotemporally resolved, contextualized, and personal exposure, behavior, and outcome information. The BREATHE Kit was designed to minimize recall bias in the outcome and behavior data, ascertain context, and reduce exposure measurement error. GPS tracking and integration of modeled environmental exposure data also enabled us to expand the suite of exposures that could be investigated and provided a robust sample size for detecting associations, compared with personal measurements that had the highest degree of missingness. As sensors continue to advance, we expect battery life and burden issues to continue to improve, allowing longer deployments and more complete data streams.

In conclusion, we found evidence of daily air pollution effects on lung function, rescue inhaler use, and feeling scared of having trouble breathing because of asthma using a longitudinal panel design. Our study provides further support for considering the importance of air pollution in the management and treatment of asthma. Our study demonstrates how informatics platforms such as our Los Angeles PRISMS Center BREATHE Kit can enable researchers to collect and integrate highly resolved measured and modeled data for investigating acute triggers of pediatric asthma or risk factors for other chronic diseases. Future directions of research include incorporating activity space-based exposures to capture conditions within actual spaces participants visited throughout the day and investigate their relationships with different metrics of personal exposures, as well as investigating within-day, within-person associations between exposures, behaviors, and health outcomes in context.

## Figures and Tables

**Table 1:** Descriptive statistics of participant characteristics (*n* = 40).

Characteristics	Statistics
Age (years, mean (range))	12.0 (8.4–16.8)
Sex (*n* (%))	
Female	18 (45.0)
Male	22 (55.0)
Race (*n* (%))	
White	15 (37.5)
Black/African American	2 (5.0)
Black/Not African American	1 (2.5)
Asian	4 (10.0)
Other	15 (37.5)
Missing	3 (7.5)
Hispanic Ethnicity (*n* (%))	
No	19 (47.5)
Yes	17 (42.5)
Missing	4 (10.0)
Caretaker’s highest completed educational grade (*n* (%))	
High school or GED	3 (7.5)
Some college or trade school	9 (22.5)
College	9 (22.5)
Graduate school	15 (37.5)
Missing	4 (10.0)
Total household income per year (*n* (%))	
Prefer not to say	12 (30)
USD 30,000–40,000	2 (5.0)
Over USD 50,000	23 (57.5)
Missing	3 (7.5)
Type of Health Insurance (*n* (%))	
HMO	18 (45.0)
PPO or POS	20 (50.0)
Missing	2 (5.0)

**Table 2:** Distributions of outcomes on the person-day level, including lung function, inhaler medication use, and asthma symptoms.

Lung Function	Mean ± SD
Percent-predicted FEV_1_ (%)	
Morning (*n* = 175)	67.9 ± 17.3
Evening (*n* = 147)	70.9 ± 17.7
Daily Average (*n* = 96)	68.7 ± 15.7
Percent-predicted PEF (%)	
Morning (*n* = 175)	69.1 ± 18.4
Evening (*n* = 147)	73.8 ± 18.3
Daily Average (*n* = 96)	69.3 ± 15.8
**Inhaler Medication**	**Mean ± SD**
Number of rescue inhaler puffs per day (*n* = 324)	1.4 ± 3.5
Number of control inhaler puffs per day (*n* = 312)	1.5 ± 1.9
**Asthma Symptoms**	***n* (%)**
Did you wake up last night because of your asthma?	
No	123 (93.2)
Yes	9 (6.8)
How many times did you use your inhaler during the night?	
Never	111 (84.1)
One or more times	21 (15.9)
How much of the time did your asthma keep you from getting as much done at school or at home today?	
Not at all	94 (86.2)
A little/Quite a bit/Very much so	15 (13.8)
Did your chest feel tight because of asthma today?	
Not at all	138 (63.6)
A little/Quite a bit/Very much so	79 (26.4)
Did you feel wheezy because of your asthma today?	
Not at all	179 (82.5)
A little/Quite a bit/Very much so	38 (17.5)
Did you have trouble breathing because of your asthma today?	
Not at all	147 (67.7)
A little/Quite a bit/Very much so	70 (32.3)
Did you cough because of your asthma today?	
Not at all	124 (57.1)
A little/Quite a bit/Very much so	93 (42.9)
How much of a problem was your asthma when you ran, exercised, or played sports today?	
Not at all	79 (71.8)
A little/Quite a bit/Very much so	31 (28.2)
In the past hour, did you feel scared that you might have trouble breathing because of your asthma?	
Not at all	152 (81.7)
A little/Quite a bit/Very much so	34 (18.3)
In the past hour, have you avoided strenuous activities, or had to slow down or stop exercising because of your asthma?	
Not at all	155 (83.3)
A little/Quite a bit/Very much so	31 (16.7)

## Supplementary Materials

The following supporting information can be downloaded at: https://www.mdpi.com/article/10.3390/ijerph19063578/s1, Table S1. Descriptive statistics of participants characteristics (*n* = 40); Table S2. Descriptive statistics of daily (6 a.m.–6 a.m.) air pollution exposures and meteorology; Table S3. Exposure (daily average, 6 a.m.–6 a.m.) Spearman correlation matrix; Table S4. Change in %predicted morning, evening or daily averaged FEV1 (forced expiratory volume in 1 s) per standard deviation change in pollutant exposure; Table S5. Change in %predicted morning, evening or daily PEF (peak expiratory flow rate) (per standard deviation change) in pollutant exposure; Table S6. Change in %predicted morning, evening or daily FEV1 (forced expiratory volume in 1 s) per standard deviation change in pollutant exposure on lag 0 day adjusted personal relative humidity, Hispanic ethnicity, and daily control inhaler use; Table S7. Change in %predicted morning, evening or daily PEF (peak expiratory flow rate) (per standard deviation change) in pollutant exposure on lag 0 day adjusted personal relative humidity, Hispanic ethnicity, and daily control inhaler use; Table S8. Associations between daily air pollutant exposures and count of rescue inhaler puffs used (rate ratios per standard deviation change in pollutant); Table S9. Associations between same-day (lag 0) daily air pollutant exposures and asthma symptoms (odds ratio per standard deviation change in pollutant); Table S10. Associations between same-day (lag 0) personal PM_2.5_ and ambient O_3_ in models adjusted for personal relative humidity, Hispanic ethnicity, Parental asthma status, and Caretaker education (fully adjusted) versus models adjusted for personal relative humidity and Hispanic ethnicity (final models as presented in main analysis). Effects are on original scale per unit change in pollutant, for morning FEV_1_ lung function, chest feeling tight symptom, and rescue inhaler use outcomes to demonstrate the impact of adjustments on final reported effects; Figure S1. Comparison between forced expiratory volume in one second (FEV1) collected at recruitment with the Asma-1 BT sensor used in the BREATHE Kit and the and clinic spirometer tested FEV_1_ on same or closest previous day to recruitment (units of L/s); Figure S2. Rescue inhaler use daily count distribution across all subjects; Figure S3. Distribution of daily average personal (measured), ambient (modeled) and traffic-related (modeled) PM_2.5_ exposures in µg/m^3^ across all subjects; Figure S4. Results of single- and two-pollutant models (rate ratio and 95% CI per standard deviation increase in exposure) for daily count of rescue inhaler use in relation to traffic-related PM_2.5_, NO, and NO_x_ exposure in the last 24 h (lag 0).
