# Supplementary material for: Daily Associations of Air Pollution and Pediatric Asthma Risk Using the Biomedical REAI-Time Health Evaluation (BREATHE) Kit
Source: Int J Environ Res Public Health. 2022 Mar 17;19(6):3578. doi: 10.3390/ijerph19063578 (PMC8950308; doi:10.3390/ijerph19063578)

SUPPLEMENT

Daily Associations of Air Pollution and Pediatric Asthma Risk using the Biomedical REAl-Time Health Evaluation (BREATHE) Kit

Hua Hao, PhD[1], Sandrah P. Eckel, PhD[1], Anahita Hosseini, PhD[2], Eleanne D.S. Van Vliet, DrPH[3], Eldin Dzubur, PhD[1], Genevieve Dunton, PhD[1], Shih Ying Chang, PhD[4], Kenneth Craig[4], Rose Rocchio, PhD[5], Theresa Bastain, PhD[1], Frank Gilliland, MD, PhD[1], Sande Okelo, PhD[6], Mindy K. Ross, MD[6], Majid Sarrafzadeh, PhD[2], Alex A. T. Bui, PhD[7], Rima Habre, ScD[1]

[1] Department of Population and Public Health Sciences, University of Southern California, Los Angeles, CA, USA
[2] Department of Computer Science, University of California Los Angeles, Los Angeles, CA
[3] Health Effects Institute, Boston, MA
[4] Sonoma Technology, Inc., Petaluma, CA
[5] Mobilize Labs, University of California Los Angeles, Los Angeles, CA
[6] Department of Pediatrics, University of California Los Angeles, Los Angeles, CA
[7] Medical & Imaging Informatics Group, Department of Radiological Sciences, University of California Los Angeles, Los Angeles, CA

TABLE OF CONTENTS

Supplement Table 1. Descriptive statistics of participants characteristics (N=40). 3

Supplement Table 2. Descriptive statistics of daily (6am-6am) air pollution exposures and meteorology. 5

Supplement Table 3. Exposure (daily average, 6am-6am) Spearman correlation matrix 6

Supplement Table 4. Change in %predicted morning, evening or daily averaged FEV1 (forced expiratory volume in 1 second) per standard deviation change in pollutant exposure. 7

Supplement Table 5. Change in %predicted morning, evening or daily PEF (peak expiratory flow rate) (per standard deviation change) in pollutant exposure. 9

Supplement Table 6. Change in %predicted morning, evening or daily FEV_1 (forced expiratory volume in 1 second) per standard deviation change in pollutant exposure on lag 0 day adjusted personal relative humidity, Hispanic ethnicity, and daily control inhaler use. ... 11

Supplement Table 7. Change in %predicted morning, evening or daily PEF (peak expiratory flow rate) (per standard deviation change) in pollutant exposure on lag 0 day adjusted personal relative humidity, Hispanic ethnicity, and daily control inhaler use. 12

Supplement Table 8. Associations between daily air pollutant exposures and count of rescue inhaler puffs used (rate ratios per standard deviation change in pollutant). 13

Supplement Table 9. Associations between same-day (lag 0) daily air pollutant exposures and asthma symptoms (odds ratio per standard deviation change in pollutant). 14

Supplement Table 10. Associations between same-day (lag 0) personal $PM_{2.5}$ and ambient O_3 in models adjusted for personal relative humidity, Hispanic ethnicity, Parental asthma status, and Caretaker education (fully adjusted) versus models adjusted for personal relative humidity and Hispanic ethnicity (final models as presented in main analysis). Effects are on original

scale per unit change in pollutant, for morning FEV_1 lung function, chest feeling tight symptom, and rescue inhaler use outcomes to demonstrate the impact of adjustments on final reported effects. 17

Supplement Figure 1. Comparison between forced expiratory volume in one second (FEV_1) collected at recruitment with the Asma-1 BT sensor used in the BREATHE Kit and the and clinic spirometer tested FEV_1 on same or closest previous day to recruitment (units of L/s). . 20

Supplement Figure 2. Rescue inhaler use daily count distribution across all subjects. 21

Supplement Figure 3. Distribution of daily average personal (measured), ambient (modeled) and traffic-related (modeled) $PM_{2.5}$ exposures in µg/m³ across all subjects. 22

Supplement Figure 4. Results of single- and two-pollutant models (rate ratio and 95% CI per standard deviation increase in exposure) for daily count of rescue inhaler use in relation to traffic-related $PM_{2.5}$, NO, and NO_x exposure in the last 24 hours (lag 0). 23

Supplement Table S1. Descriptive statistics of participants characteristics (N=40).

**Characteristics**	**Statistics**
Medications taken in the last 12 months	
Short-acting beta$_2$-agonist bronchodilators [N (%)]	
No	11 (27.5)
Regularly, every day	4 (10.0)
Occasionally, as needed	25 (62.5)
Long-acting beta$_2$-agonist bronchodilators [N (%)]	
No	40 (100.0)
Corticosteroids [N (%)]	
No	28 (70.0)
Regularly, every day	12 (30.0)
Oral steroid medication [N (%)]	
No	25 (62.5)
Yes	15 (37.5)
Have allergies [N (%)]	
No	8 (20.0)
Yes	27 (67.5)
Missing	5 (12.5)
Any biological parents ever been diagnosed with asthma [N (%)]	
No	25 (62.5)
Yes	10 (25.0)
Missing	5 (12.5)
Anyone currently smoke cigarettes, or anything other than cigarettes (e-cigarettes, cigars, pipes, hookah's, tobacco products, other) at the home on a regular basis [N (%)]	
No	37 (92.5)
Missing	3 (7.5)
Child ever smoked electronic cigarettes/e-cigarettes or other electronic nicotine devices (e-hookah, e-cigars, etc.) [N (%)]	
No	34 (85.0)
Missing	6 (15.0)
Mother have any problems during the pregnancy [N (%)]	
An infection	1 (2.5)
Early labor	7 (17.5)
High blood pressure	2 (5.0)
High blood sugar	6 (15.0)
None of these	18 (45.0)
Missing	6 (15.0)

Mother use during pregnancy [N (%)]	
Cigarettes	0 (0)
Drugs	1 (2.5)
None of these	36 (90)
Missing	3 (7.5)
Kitchen fan over the cooking stove, range, oven or elsewhere in the kitchen [N (%)]	
No	5 (12.5)
Yes	32 (80.0)
Missing	3 (7.5)
Child own pets [N (%)]	
No	15 (37.5)
Yes	22 (55.0)
Missing	3 (7.5)
Any sports in a typical week [N (%)]	
No	7 (17.5)
Yes	29 (72.5)
Missing	4 (10.0)
Days play outdoors for at least half an hour in a typical week [N (%)]	
No	6 (15.0)
Yes	31 (77.5)
Missing	3 (7.5)

Supplement Table S2. Descriptive statistics of daily (6am-6am) air pollution exposures and meteorology.

Environmental exposure	N (person-days)	Mean	Std Dev	Min - Max
Personal Exposure (measured)				
$PM_{2.5}$ (μg/m^3)	182	6.9	9.1	0.3 - 64.7
Temperature (℃)	182	27.4	2.2	22.5 - 33.2
Relative Humidity (%)	182	45.9	8.1	15.3 - 61.1
Ambient Pollutants (modeled)				
$PM_{2.5}$ (μg/m^3)	359	9.5	3.7	1.0 - 21.1
NO_2 (ppb)	356	7.6	4.6	0.27 - 35.9
NO_x (ppb)	345	9.5	6.1	0.27 - 36.0
NO (ppb)	345	1.8	2.4	0 - 17.3
O_3 (ppb)	359	32.3	9.2	7.7 - 64.7
Temperature (℃)	342	18.0	4.0	8.2 - 32.2
Relative Humidity (%)	342	69.7	19.1	8.2 - 95.8
Traffic-related Pollutants (modeled)				
$PM_{2.5}$ (μg/m^3)	338	0.7	0.7	0.1 - 4.8
NO_2 (ppb)	338	4.9	7.1	0 - 42.8
NO_x (ppb)	338	10.6	14.7	0.1 - 88.9

Supplement Table S3. Exposure (daily average, 6am-6am) Spearman correlation matrix

	Personal	Ambient					Traffic-related		
	$PM_{2.5}$	NO_2	NO_x	NO	O_3	$PM_{2.5}$	NO_2	NO_x	$PM_{2.5}$
Personal $PM_{2.5}$	1.00	0.05	-0.03	-0.03	-0.10	0.39*	0.15	0.14	0.14
Ambient NO_2		1.00	0.98*	0.67*	-0.40*	0.21*	0.19*	0.20*	0.24*
Ambient NO_x			1.00	0.78*	-0.37*	0.18*	0.16*	0.17*	0.21*
Ambient NO				1.00	-0.25*	0.24*	-0.05	-0.03	-0.01
Ambient O_3					1.00	-0.03	-0.22*	-0.23*	-0.24*
Ambient $PM_{2.5}$						1.00	0.09	0.08	0.09
Traffic-related NO_2							1.00	0.99*	0.98*
Traffic-related NO_x								1.00	0.99*
Traffic-related $PM_{2.5}$									1.00

*P<0.05 from Wald-based tests of Spearman correlation coefficient

Supplement Table S4. Change in %predicted morning, evening or daily averaged FEV1 (forced expiratory volume in 1 second) per standard deviation change in pollutant exposure.

Exposure	Morning %Predicted FEV_1 Estimate (95% CI)	P-value	N (person-days)
Personal $PM_{2.5}$ (μg/m^3)			
Lag 0#	2.89 (-0.18, 5.96)	0.070	86
Lag 1#	-0.21 (-3.89, 3.47)	0.912	72
Lag 2#	-2.22 (-6.63, 2.19)	0.329	63
Traffic-related $PM_{2.5}$ (μg/m^3)			
Lag 0	-1.50 (-5.03, 2.03)	0.408	123
Lag 1	**-3.91 (-7.36, -0.47)**	**0.029**	112
Lag 2	-5.57 (-12.22, 1.08)	0.105	95
Traffic-related NO_x (ppb)			
Lag 0	-1.08 (-5.19, 3.02)	0.607	123
Lag 1	**-5.05 (-9.32, -0.78)**	**0.023**	112
Lag 2	-5.38 (-12.74, 1.98)	0.156	95
Traffic-related NO_2 (ppb)			
Lag 0	-0.38 (-4.10, 3.34)	0.843	123
Lag 1	**-4.81 (-8.65, -0.97)**	**0.016**	112
Lag 2	-4.75 (-11.70, 2.21)	0.185	95
Ambient $PM_{2.5}$ (μg/m^3)			
Lag 0	-2.60 (-5.96, 0.76)	0.133	132
Lag 1	-1.18 (-4.75, 2.38)	0.517	116
Lag 2	-0.25 (-3.93, 3.43)	0.896	101
Ambient O_3 (ppb)			

Exposure	Evening %Predicted FEV_1 Estimate (95% CI)	P-value	N (person-days)
Personal $PM_{2.5}$ (μg/m^3)			
Lag 0#	-1.31 (-4.37, 1.75)	0.405	74
Lag 1#	-0.04 (-3.47, 3.39)	0.982	68
Lag 2#	8.21 (-11.22, 27.63)	0.412	57
Traffic-related $PM_{2.5}$ (μg/m^3)			
Lag 0	-2.55 (-5.64, 0.54)	0.110	101
Lag 1	1.59 (-2.33, 5.50)	0.430	88
Lag 2	0.94 (-2.76, 4.64)	0.619	77
Traffic-related NO_x (ppb)			
Lag 0	-2.56 (-5.98, 0.86)	0.146	101
Lag 1	1.10 (-3.07, 5.26)	0.607	88
Lag 2	1.12 (-2.95, 5.20)	0.591	77
Traffic-related NO_2 (ppb)			
Lag 0	-2.21 (-5.33, 0.91)	0.168	101
Lag 1	0.98 (-2.78, 4.75)	0.611	88
Lag 2	1.06 (-2.65, 4.77)	0.577	77
Ambient $PM_{2.5}$ (μg/m^3)			
Lag 0	2.03 (-0.71, 4.77)	0.150	110
Lag 1	-2.43 (-6.18, 1.31)	0.207	96
Lag 2	-0.96 (-4.36, 2.45)	0.584	84
Ambient O_3 (ppb)			

Exposure	Daily %Predicted FEV_1 Estimate (95% CI)	P-value	N (person-days)
Personal $PM_{2.5}$ (μg/m^3)			
Lag 0#	-1.81 (-4.40, 0.79)	0.182	52
Lag 1#	0.33 (-2.72, 3.38)	0.832	46
Lag 2#	11.44 (-13.28, 36.16)	0.373	39
Traffic-related $PM_{2.5}$ (μg/m^3)			
Lag 0	1.06 (-6.29, 8.42)	0.778	69
Lag 1	-1.49 (-5.56, 2.59)	0.478	62
Lag 2	-3.00 (-6.64, 0.63)	0.114	55
Traffic-related NO_x (ppb)			
Lag 0	1.92 (-7.86, 11.71)	0.702	69
Lag 1	-1.81 (-5.97, 2.35)	0.399	62
Lag 2	-2.91 (-6.83, 1.01)	0.153	55
Traffic-related NO_2 (ppb)			
Lag 0	3.03 (-6.48, 12.53)	0.535	69
Lag 1	-1.60 (-5.30, 2.11)	0.402	62
Lag 2	-2.60 (-6.15, 0.95)	0.159	55
Ambient $PM_{2.5}$ (μg/m^3)			
Lag 0	1.55 (-1.62, 4.73)	0.342	76
Lag 1	-1.07 (-4.86, 2.72)	0.582	66
Lag 2	0.55 (-2.87, 3.96)	0.754	59
Ambient O_3 (ppb)			

Lag 0	**-4.11 (-6.86, -1.36)**	**0.004**	132	**-2.65 (-5.19, -0.10)**	**0.045**	110	**-3.45 (-6.42, -0.47)**	**0.027**	76
Lag 1	-1.25 (-4.13, 1.62)	0.395	116	**-4.90 (-7.94, -1.85)**	**0.002**	96	**-4.92 (-8.44, -1.40)**	**0.009**	66
Lag 2	**-2.94 (-5.93, 0.05)**	**0.057**	101	-1.16 (-4.45, 2.12)	0.490	84	0.90 (-3.27, 5.07)	0.674	59
Ambient NO_x (ppb)									
Lag 0	-0.34 (-3.79, 3.11)	0.849	125	2.11 (-1.06, 5.28)	0.196	102	2.70 (-1.24, 6.64)	0.185	70
Lag 1	-2.18 (-5.67, 1.31)	0.225	113	2.76 (-1.23, 6.75)	0.179	89	-0.96 (-5.33, 3.42)	0.671	63
Lag 2	-0.73 (-4.58, 3.12)	0.712	96	1.84 (-2.99, 6.67)	0.458	78	-1.10 (-6.81, 4.61)	0.708	55
Ambient NO (ppb)									
Lag 0	-0.52 (-4.29, 3.25)	0.786	125	2.64 (-0.77, 6.05)	0.133	102	2.17 (-1.80, 6.14)	0.289	70
Lag 1	-2.10 (-5.71, 1.52)	0.259	113	1.76 (-2.10, 5.61)	0.375	89	-0.93 (-4.79, 2.94)	0.641	63
Lag 2	-0.70 (-4.66, 3.25)	0.729	96	-0.07 (-6.14, 6.00)	0.982	78	-1.55 (-8.64, 5.55)	0.672	55
Ambient NO_2 (ppb)									
Lag 0	0.04 (-2.80, 2.87)	0.981	130	1.90 (-1.23, 5.04)	0.238	107	2.33 (-1.63, 6.28)	0.254	74
Lag 1	-2.03 (-5.70, 1.63)	0.280	115	3.15 (-0.94, 7.24)	0.136	94	-0.79 (-5.32, 3.74)	0.735	65
Lag 2	-0.96 (-4.71, 2.79)	0.616	100	2.07 (-2.33, 6.47)	0.361	82	-1.52 (-6.72, 3.68)	0.570	58

*Effect estimates were scaled to a standard deviation change in pollutant concentrations as follows: personal $PM_{2.5}$: 9.1 μg/m^3; Traffic-related $PM_{2.5}$: 0.7 μg/m^3; Traffic-related NO_x: 14.7 ppb; Traffic-related NO_2: 7.1 ppb; Ambient $PM_{2.5}$: 3.7 μg/m^3; Ambient O_3: 9.2 ppb; Ambient NO_x: 6.1 ppb; Ambient NO: 2.4 ppb; Ambient NO_2: 4.6 ppb.

#Lag 0 was defined as preceding 24 hours and lags 1 and 2 days were defined as the average of the 25th to 48th hour (lag 1) and the average of the 49th to 72nd hour (lag 2).

Supplement Table S5. Change in %predicted morning, evening or daily PEF (peak expiratory flow rate) (per standard deviation change) in pollutant exposure.

	**Morning %Predicted PEF**			**Evening %Predicted PEF**			**Daily %Predicted PEF**		
**Exposure**	**Estimate (95% CI)**	**P=value**	**N (person -days)**	**Estimate (95% CI)**	**P-value**	**N (person -days)**	**Estimate (95% CI)**	**P-value**	**N (person -days)**
Personal $PM_{2.5}$ ($\mu g/m^3$)									
Lag 0[#]	0.94 (-2.15, 4.03)	0.553	86	-1.91 (-5.33, 1.51)	0.278	74	-2.31 (-5.07, 0.46)	0.112	52
Lag 1[#]	-1.03 (-4.56, 2.50)	0.570	72	-0.07 (-4.26, 4.11)	0.973	68	-1.09 (-4.38, 2.20)	0.520	46
Lag 2[#]	-2.72 (-7.06, 1.61)	0.225	63	1.73 (-17.05, 20.52)	0.857	57	4.28 (-19.51, 28.08)	0.727	39
Traffic $PM_{2.5}$ ($\mu g/m^3$)									
Lag 0	**-3.97 (-7.69, -0.26)**	**0.039**	123	-2.59 (-5.92, 0.74)	0.131	101	-6.68 (-14.32, 0.96)	0.093	69
Lag 1	-3.35 (-6.89, 0.19)	0.067	112	-0.24 (-4.37, 3.90)	0.911	88	-1.30 (-5.49, 2.88)	0.544	62
Lag 2	-6.27 (-12.75, 0.21)	0.062	95	-1.24 (-4.64, 2.15)	0.475	77	-2.99 (-6.36, 0.38)	0.090	55
Traffic NO_x (ppb)									
Lag 0	-3.59 (-7.92, 0.74)	0.108	123	-2.45 (-6.12, 1.23)	0.196	101	-7.76 (-17.99, 2.48)	0.144	69
Lag 1	**-4.91 (-9.28, -0.54)**	**0.030**	112	-0.74 (-5.11, 3.64)	0.743	88	-1.54 (-5.79, 2.72)	0.483	62
Lag 2	-6.04 (-13.22, 1.14)	0.104	95	-0.99 (-4.73, 2.75)	0.606	77	-2.81 (-6.43, 0.80)	0.136	55
Traffic NO_2 (ppb)									
Lag 0	-2.75 (-6.69, 1.20)	0.175	123	-2.11 (-5.46, 1.24)	0.220	101	-6.61 (-16.60, 3.39)	0.201	69
Lag 1	**-4.57 (-8.51, -0.63)**	**0.026**	112	-0.58 (-4.54, 3.38)	0.774	88	-1.32 (-5.09, 2.45)	0.497	62
Lag 2	-5.44 (-12.22, 1.33)	0.120	95	-0.82 (-4.22, 2.57)	0.637	77	-2.55 (-5.82, 0.72)	0.135	55
Ambient $PM_{2.5}$ ($\mu g/m^3$)									
Lag 0	-2.40 (-5.96, 1.16)	0.190	132	2.48 (-0.41, 5.37)	0.096	110	0.37 (-3.11, 3.86)	0.835	76
Lag 1	-0.27 (-3.80, 3.27)	0.883	116	-0.69 (-4.60, 3.23)	0.731	96	-0.96 (-4.71, 2.79)	0.617	66
Lag 2	-0.70 (-4.21, 2.81)	0.697	101	-0.52 (-3.69, 2.65)	0.749	84	0.65 (-2.55, 3.85)	0.692	59
Ambient O_3 (ppb)									
Lag 0	-1.96 (-4.97, 1.05)	0.205	132	-1.09 (-3.86, 1.67)	0.440	110	-2.19 (-5.51, 1.14)	0.203	76
Lag 1	-0.80 (-3.65, 2.05)	0.583	116	-1.96 (-5.28, 1.37)	0.252	96	-4.13 (-7.69, 0.57)	0.102	66
Lag 2	-2.22 (-5.03, 0.59)	0.126	101	-0.49 (-3.56, 2.58)	0.756	84	0.42 (-3.46, 4.30)	0.834	59

Ambient NO_x (ppb)									
Lag 0	-0.22 (-3.85, 3.41)	0.906	125	1.08 (-2.33, 4.49)	0.537	102	2.21 (-1.98, 6.40)	0.306	70
Lag 1	-0.21 (-3.71, 3.29)	0.907	113	0.56 (-3.70, 4.81)	0.798	89	-0.12 (-4.64, 4.40)	0.958	63
Lag 2	-0.68 (-4.43, 3.07)	0.724	96	1.80 (-2.69, 6.29)	0.435	78	3.63 (-1.65, 8.91)	0.185	55
Ambient NO (ppb)									
Lag 0	0.10 (-3.86, 4.06)	0.959	125	1.94 (-1.74, 5.62)	0.305	102	1.41 (-2.82, 5.63)	0.517	70
Lag 1	0.33 (-3.30, 3.95)	0.860	113	0.06 (-4.02, 4.14)	0.978	89	-0.79 (-4.71, 3.12)	0.693	63
Lag 2	-0.16 (-4.07, 3.74)	0.935	96	0.26 (-5.40, 5.92)	0.928	78	3.87 (-2.80, 10.54)	0.262	55
Ambient NO_2 (ppb)									
Lag 0	-0.25 (-3.24, 2.75)	0.872	130	0.81 (-2.55, 4.18)	0.637	107	2.21 (-2.02, 6.44)	0.310	74
Lag 1	-0.48 (-4.13, 3.18)	0.798	115	1.29 (-3.04, 5.61)	0.561	94	0.78 (-3.95, 5.50)	0.749	65
Lag 2	-0.53 (-4.18, 3.11)	0.776	100	2.24 (-1.86, 6.34)	0.289	82	2.91 (-2.08, 7.90)	0.260	58

*Effect estimates were scaled to a standard deviation change in pollutant concentrations as follows: personal $PM_{2.5}$: 9.1 $\mu g/m^3$; Traffic-related $PM_{2.5}$: 0.7 $\mu g/m^3$; Traffic-related NO_x: 14.7 ppb; Traffic-related NO_2: 7.1 ppb; Ambient $PM_{2.5}$: 3.7 $\mu g/m^3$; Ambient O_3: 9.2 ppb; Ambient NO_x: 6.1 ppb; Ambient NO: 2.4 ppb; Ambient NO_2: 4.6 ppb.

[#]Lag 0 was defined as preceding 24 hours and lags 1 and 2 days were defined as the average of the 25th to 48th hour (lag 1) and the average of the 49th to 72nd hour (lag 2).

Supplement Table S6. Change in %predicted morning, evening or daily FEV_1 (forced expiratory volume in 1 second) per standard deviation change in pollutant exposure on lag 0 day adjusted personal relative humidity, Hispanic ethnicity, and daily control inhaler use.

	**Morning %Predicted FEV_1**			**Evening %Predicted FEV_1**			**Average Daily %Predicted FEV_1**		
	**Estimate**	**95% CI**	**P-value**	**Estimate**	**95% CI**	**P-value**	**Estimate**	**95% CI**	**P-value**
Personal $PM_{2.5}$ (μg/m^3)	2.32	-1.05, 5.68	0.183	-1.78	-6.50, 2.94	0.464	-3.57	-8.33, 1.20	0.153
Traffic-related $PM_{2.5}$ (μg/m^3)	0.26	-4.03, 4.55	0.905	-1.00	-4.92, 2.91	0.618	0.36	-4.72, 5.43	0.891
Traffic-related NO_x (ppb)	0.48	-4.02, 4.97	0.836	-1.49	-5.58, 2.60	0.479	-0.22	-5.95, 5.52	0.941
Traffic-related NO_2 (ppb)	2.24	-2.08, 6.55	0.314	-1.19	-5.30, 2.92	0.572	1.02	-4.76, 6.81	0.732
Ambient $PM_{2.5}$ (μg/m^3)	-3.47	-6.95, 0.01	0.056	1.6	-2.31, 5.51	0.426	-1.72	-6.04, 2.61	0.443
Ambient O_3 (ppb)	**-4.73**	**-7.98, -1.47**	**0.006**	-2.61	-6.22, 1.00	0.163	-3.19	-7.46, 1.09	0.155
Ambient NO_x (ppb)	-0.87	-4.98, 3.25	0.682	1.1	-3.50, 5.70	0.642	2.26	-3.65, 8.18	0.460
Ambient NO (ppb)	-2.22	-6.17, 1.74	0.277	1.41	-3.14, 5.97	0.547	0.65	-5.46, 6.77	0.836
Ambient NO_2 (ppb)	-0.09	-4.03, 3.84	0.963	0.83	-3.81, 5.48	0.727	2.48	-3.22, 8.19	0.401

*Effect estimates were scaled to a standard deviation change in pollutant concentrations as follows: personal $PM_{2.5}$: 9.1 μg/m^3; Traffic-related $PM_{2.5}$: 0.7 μg/m^3; Traffic-related NO_x: 14.7 ppb; Traffic-related NO_2: 7.1 ppb; Ambient $PM_{2.5}$: 3.7 μg/m^3; Ambient O_3: 9.2 bbp; Ambient NO_x: 6.1 ppb; Ambient NO: 2.4 ppb; Ambient NO_2: 4.6

Supplement Table S7. Change in %predicted morning, evening or daily PEF (peak expiratory flow rate) (per standard deviation change) in pollutant exposure on lag 0 day adjusted personal relative humidity, Hispanic ethnicity, and daily control inhaler use.

	**Morning %Predicted PEF**			**Evening %Predicted PEF**			**Averaged Daily %Predicted PEF**		
	Estimate	**95%CI**	**P-value**	**Estimate**	**95%CI**	**P-value**	**Estimate**	**95%CI**	**P-value**
Personal $PM_{2.5}$ ($\mu g/m^3$)	0.33	-3.13, 3.78	0.854	-2.24	-7.51, 3.03	0.409	-3.63	-8.67, 1.41	0.169
Traffic-related $PM_{2.5}$ ($\mu g/m^3$)	-3.42	-7.66, 0.83	0.121	-3.83	-8.05, 0.40	0.083	**-4.89**	**-9.21, -0.56**	**0.037**
Traffic-related NO_x (ppb)	-2.60	-7.06, 1.86	0.259	-4.41	-8.81, -0.01	0.056	**-5.80**	**-10.70, -0.91**	**0.029**
Traffic-related NO_2 (ppb)	-1.52	-5.85, 2.81	0.495	-4.31	-8.73, 0.12	0.063	-5.19	-10.27, -0.11	0.057
Ambient $PM_{2.5}$ ($\mu g/m^3$)	-1.98	-5.63, 1.67	0.292	3.15	-1.10, 7.41	0.153	-2.14	-6.47, 2.19	0.342
Ambient O_3 (ppb)	-2.31	-5.82, 1.20	0.202	-1.03	-5.09, 3.04	0.623	-4.30	-8.55, -0.05	0.057
Ambient NO_x (ppb)	0.07	-4.03, 4.17	0.975	0.05	-5.19, 5.29	0.985	1.43	-4.98, 7.85	0.666
Ambient NO (ppb)	-0.22	-4.20, 3.75	0.912	2.10	-3.09, 7.28	0.433	0.24	-6.61, 7.08	0.946
Ambient NO_2 (ppb)	-0.20	-4.24, 3.84	0.923	-0.87	-6.08, 4.35	0.746	2.00	-3.82, 7.82	0.506

*Effect estimates were scaled to a standard deviation change in pollutant concentrations as follows: personal $PM_{2.5}$: 9.1 $\mu g/m^3$; Traffic-related $PM_{2.5}$: 0.7 $\mu g/m^3$; Traffic-related NO_x: 14.7 ppb; Traffic-related NO_2: 7.1 ppb; Ambient $PM_{2.5}$: 3.7 $\mu g/m^3$; Ambient O_3: 9.2 bbp; Ambient NO_x: 6.1 ppb; Ambient NO: 2.4 ppb; Ambient NO_2: 4.6 ppb

Supplement Table S8. Associations between daily air pollutant exposures and count of rescue inhaler puffs used (rate ratios per standard deviation change in pollutant).

Exposure	Rate Ratio (95% CI)	P-value	N (person-days)
Personal $PM_{2.5}$ (μg/m^3)			
Lag 0#	1.09 (0.80, 1.50)	0.580	86
Lag 1#	1.34 (0.77, 2.35)	0.306	81
Lag 2#	1.16 (0.91, 1.47)	0.238	77
Traffic $PM_{2.5}$ (μg/m^3)			
Lag 0	**0.48 (0.26, 0.88)**	**0.021**	166
Lag 1	0.68 (0.30, 1.53)	0.352	154
Lag 2	0.56 (0.23, 1.39)	0.217	143
Traffic NO_x (ppb)			
Lag 0	**0.33 (0.13, 0.84)**	**0.023**	166
Lag 1	0.47 (0.13, 1.72)	0.261	154
Lag 2	0.48 (0.12, 1.92)	0.307	143
Traffic NO_2 (ppb)			
Lag 0	**0.34 (0.14, 0.84)**	**0.022**	166
Lag 1	0.50 (0.15, 1.71)	0.276	154
Lag 2	0.74 (0.22, 2.45)	0.624	143
Ambient $PM_{2.5}$ (μg/m^3)			
Lag 0	1.45 (0.90, 2.33)	0.127	167
Lag 1	0.78 (0.42, 1.47)	0.448	155
Lag 2	2.56 (0.89, 4.71)	0.187	144
Ambient O_3 (ppb)			
Lag 0	**1.52 (1.02, 2.27)**	**0.046**	167
Lag 1	0.99 (0.59, 1.68)	0.982	155
Lag 2	0.80 (0.48, 1.32)	0.384	144
Ambient NO_x (ppb)			
Lag 0	**1.61 (1.23, 2.11)**	**0.001**	166
Lag 1	0.79 (0.59, 1.07)	0.134	155
Lag 2	0.42 (0.26, 1.09)	0.145	144
Ambient NO (ppb)			
Lag 0	**1.80 (1.37, 2.35)**	**0.000**	166
Lag 1	1.01 (0.85, 1.21)	0.902	155
Lag 2	0.45 (0.17, 1.33)	0.208	144
Ambient NO_2 (ppb)			
Lag 0	1.20 (0.85, 1.68)	0.302	166
Lag 1	0.46 (0.29, 1.12)	0.198	155
Lag 2	0.45 (0.28, 1.11)	0.186	144

*Effect estimates were scaled to a standard deviation change in pollutant concentrations as follows: personal $PM_{2.5}$: 9.1 μg/m^3; Traffic-related $PM_{2.5}$: 0.7 μg/m^3; Traffic-related NO_x: 14.7 ppb; Traffic-related NO_2: 7.1 ppb; Ambient $PM_{2.5}$: 3.7 μg/m^3; Ambient O_3: 9.2 ppb; Ambient NO_x: 6.1 ppb; Ambient NO: 2.4 ppb; Ambient NO_2: 4.6 ppb.

#Lag 0 was defined as preceding 24 hours and lags 1 and 2 days were defined as the average of the 25th to 48th hour (lag 1) and the average of the 49th to 72nd hour (lag 2).

Supplement Table S9. Associations between same-day (lag 0) daily air pollutant exposures and asthma symptoms (odds ratio per standard deviation change in pollutant).

Exposure	Estimate (95% CI)	P-value	N (person-days)
**Did you wake up last night because of your asthma?**			
Personal $PM_{2.5}$	0.71 (0.11, 4.64)	0.724	52
Traffic-related $PM_{2.5}$	0.52 (0.15, 1.85)	0.315	85
Traffic-related NO_x	0.52 (0.16, 1.70)	0.283	85
Traffic-related NO_2	0.38 (0.09, 1.56)	0.183	85
Ambient $PM_{2.5}$	1.73 (0.60, 5.00)	0.315	87
Ambient O_3	1.19 (0.43, 3.31)	0.737	87
Ambient NO_x	0.51 (0.06, 4.03)	0.525	87
Ambient NO	0.15 (0.00, 4.73)	0.285	87
Ambient NO_2	0.79 (0.14, 4.62)	0.794	87
**How many times did you use your inhaler during the night?**			
Personal $PM_{2.5}$	0.39 (0.05, 3.08)	0.379	52
Traffic-related $PM_{2.5}$	0.95 (0.37, 2.42)	0.914	85
Traffic-related NO_x	0.79 (0.33, 1.92)	0.609	85
Traffic-related NO_2	0.61 (0.22, 1.68)	0.343	85
Ambient $PM_{2.5}$	0.63 (0.23, 1.72)	0.374	87
Ambient O_3	0.97 (0.47, 1.99)	0.927	87
Ambient NO_x	0.77 (0.27, 2.22)	0.633	87
Ambient NO	1.36 (0.48, 3.87)	0.571	87
Ambient NO_2	0.52 (0.15, 1.77)	0.301	87
**How much of the time did your asthma keep you from getting as much done at school or at home today?**			
Personal $PM_{2.5}$	0.99 (0.42, 2.35)	0.986	52
Traffic-related $PM_{2.5}$	1.24 (0.50, 3.04)	0.642	81
Traffic-related NO_x	1.11 (0.53, 2.33)	0.791	81
Traffic-related NO_2	1.09 (0.52, 2.27)	0.826	81
Ambient $PM_{2.5}$	0.70 (0.25, 2.00)	0.509	83
Ambient O_3	1.13 (0.45, 2.81)	0.798	83
Ambient NO_x	1.15 (0.38, 3.43)	0.808	83
Ambient NO	1.48 (0.53, 4.14)	0.458	83
Ambient NO_2	0.95 (0.30, 2.96)	0.929	83
**Did your chest feel tight because of asthma today?**			
Personal $PM_{2.5}$	0.85 (0.51, 1.42)	0.549	92
Traffic-related $PM_{2.5}$	0.94 (0.61, 1.45)	0.796	151
Traffic-related NO_x	0.97 (0.65, 1.43)	0.874	151
Traffic-related NO_2	0.95 (0.65, 1.38)	0.794	151
Ambient $PM_{2.5}$	1.01 (0.57, 1.79)	0.969	154
Ambient O_3	0.81 (0.53, 1.25)	0.349	154
Ambient NO_x	1.24 (0.67, 2.31)	0.496	153
Ambient NO	1.13 (0.55, 2.33)	0.746	153

Ambient NO_2	1.29 (0.72, 2.30)	0.399	154
**Did you feel wheezy because of your asthma today?**			
Personal $PM_{2.5}$	0.93 (0.46, 1.88)	0.840	92
Traffic-related $PM_{2.5}$	1.37 (0.74, 2.57)	0.320	151
Traffic-related NO_x	1.17 (0.60, 2.28)	0.653	151
Traffic-related NO_2	1.16 (0.61, 2.20)	0.661	151
Ambient $PM_{2.5}$	1.08 (0.45, 2.57)	0.862	154
Ambient O_3	0.61 (0.32, 1.14)	0.122	154
Ambient NO_x	1.35 (0.63, 2.88)	0.437	153
Ambient NO	1.33 (0.57, 3.11)	0.513	153
Ambient NO_2	1.33 (0.64, 2.75)	0.444	154
**Did you have trouble breathing because of your asthma today?**			
Personal $PM_{2.5}$	0.88 (0.44, 1.75)	0.720	92
Traffic-related $PM_{2.5}$	0.85 (0.53, 1.36)	0.490	151
Traffic-related NO_x	0.73 (0.42, 1.27)	0.261	151
Traffic-related NO_2	0.73 (0.43, 1.25)	0.252	151
Ambient $PM_{2.5}$	1.00 (0.55, 1.81)	0.999	154
Ambient O_3	0.96 (0.61, 1.52)	0.875	154
Ambient NO_x	1.10 (0.58, 2.11)	0.763	153
Ambient NO	0.87 (0.40, 1.88)	0.730	153
Ambient NO_2	1.22 (0.66, 2.24)	0.529	154
**Did you cough because of your asthma today?**			
Personal $PM_{2.5}$	1.17 (0.68, 2.01)	0.581	92
Traffic-related $PM_{2.5}$	1.45 (0.84, 2.48)	0.181	151
Traffic-related NO_x	1.26 (0.74, 2.16)	0.402	151
Traffic-related NO_2	1.34 (0.80, 2.27)	0.271	151
Ambient $PM_{2.5}$	0.87 (0.47, 1.61)	0.654	154
Ambient O_3	0.66 (0.40, 1.09)	0.106	154
Ambient NO_x	1.44 (0.75, 2.76)	0.273	153
Ambient NO	1.46 (0.71, 3.03)	0.309	153
Ambient NO_2	1.37 (0.74, 2.55)	0.318	154
**How much of a problem was your asthma when you ran, exercise or play sports today?**			
Personal $PM_{2.5}$	0.66 (0.04, 10.20)	0.767	49
Traffic-related $PM_{2.5}$	1.36 (0.76, 2.45)	0.302	83
Traffic-related NO_x	1.34 (0.70, 2.58)	0.383	83
Traffic-related NO_2	1.23 (0.68, 2.24)	0.499	83
Ambient $PM_{2.5}$	1.11 (0.52, 2.39)	0.782	84
Ambient O_3	1.23 (0.62, 2.43)	0.558	84
Ambient NO_x	1.15 (0.52, 2.54)	0.731	84
Ambient NO	1.15 (0.50, 2.67)	0.746	84
Ambient NO_2	1.16 (0.53, 2.53)	0.718	84
**Did you feel scared that you might have trouble breathing because of your asthma today?**			
Personal $PM_{2.5}$	1.36 (0.70, 2.66)	0.365	85

Traffic-related $PM_{2.5}$	**1.83 (1.03, 3.24)**	**0.042**	138
Traffic-related NO_x	1.38 (0.88, 2.15)	0.163	138
Traffic-related NO_2	1.31 (0.86, 2.00)	0.206	138
Ambient $PM_{2.5}$	0.76 (0.33, 1.72)	0.507	140
Ambient O_3	1.02 (0.56, 1.84)	0.961	140
Ambient NO_x	1.74 (0.77, 3.92)	0.184	140
Ambient NO	1.35 (0.55, 3.32)	0.512	140
Ambient NO_2	1.88 (0.85, 4.19)	0.124	140
**Have you avoided strenuous activities, or had to slow down or stop exercising because of your asthma today?**			
Personal $PM_{2.5}$	0.71 (0.18, 2.84)	0.635	85
Traffic-related $PM_{2.5}$	1.12 (0.66, 1.88)	0.680	138
Traffic-related NO_x	0.98 (0.56, 1.71)	0.938	138
Traffic-related NO_2	0.96 (0.55, 1.67)	0.880	138
Ambient $PM_{2.5}$	0.67 (0.33, 1.36)	0.271	140
Ambient O_3	1.00 (0.60, 1.67)	0.986	140
Ambient NO_x	0.78 (0.38, 1.58)	0.485	140
Ambient NO	0.86 (0.37, 2.01)	0.733	140
Ambient NO_2	0.76 (0.39, 1.49)	0.424	140

*Effect estimates were scaled to a standard deviation change in pollutant concentrations as follows: personal $PM_{2.5}$: 9.1 μg/m^3; Traffic-related $PM_{2.5}$: 0.7 μg/m^3; Traffic-related NO_x: 14.7 ppb; Traffic-related NO_2: 7.1 ppb; Ambient $PM_{2.5}$: 3.7 μg/m^3; Ambient O_3: 9.2 bbp; Ambient NO_x: 6.1 ppb; Ambient NO: 2.4 ppb; Ambient NO_2: 4.6 ppb;

Supplement Table S10. Associations between same-day (lag 0) personal $PM_{2.5}$ and ambient O_3 in models adjusted for personal relative humidity, Hispanic ethnicity, Parental asthma status, and Caretaker education (fully adjusted) versus models adjusted for personal relative humidity and Hispanic ethnicity (final models as presented in main analysis). Effects are on original scale per unit change in pollutant, for morning FEV_1 lung function, chest feeling tight symptom, and rescue inhaler use outcomes to demonstrate the impact of adjustments on final reported effects.

A. Morning FEV_1

	**Fully Adjusted Model**		**Final Model**	
**Variables**	**Estimate**	**95% CI**	**Estimate**	**95% CI**
Personal $PM_{2.5}$	0.284	[-0.070, 0.638]	0.319	[-0.026, 0.665]
Relative Humidity	0.382	[-0.439, 1.203]	0.089	[-0.581, 0.760]
Hispanic Ethnicity (No)	33.5	[-23.0, 90.0]	17.1	[-7.1, 41.2]
Hispanic Ethnicity (Yes)	22.2	[-30.9, 75.3]	2.3	[-21.2, 25.9]
Hispanic Ethnicity (missing)	Reference			
Parent Asthma Status (No)	-33.2	[-80.8, 14.4]		
Parent Asthma Status (Yes)	-44.7	[-94.4, 5.1]		
Parent Asthma Status (missing)	Reference			
Caretaker education (College)	7.8	[-19.0, 34.6]		
Caretaker education (Don't know)	-4.3	[-82.3, 74.7]		
Caretaker education (Graduate School)	8.3	[-12.2, 28.7]		
Caretaker education (High School or GED)	23.0	[-27.7, 73.7]		
Caretaker education (Some college)	Reference			
Ambient O_3	-0.469	[-0.777, -0.161]	-0.448	[-0.750, -0.145]
Relative Humidity	0.201	[-0.493, 0.895]	0.130	[-0.447, 0.708]
Hispanic Ethnicity (No)	28.5	[-21.4, 78.4]	21.9	[0.1, 43.6]
Hispanic Ethnicity (Yes)	17.1	[-29.7, 63.9]	7.2	[-13.9, 28.3]
Hispanic Ethnicity (missing)	Reference			
Parent Asthma Status (No)	-6.5	[-39.3, 26.4]		
Parent Asthma Status (Yes)	-16.8	[-51.5, 18.0]		
Parent Asthma Status (missing)	Reference			
Caretaker education (College)	7.8	[-12.9, 28.6]		
Caretaker education (Don't know)	15.1	[-51.6, 81.9]		
Caretaker education (Graduate School)	11.5	[-6.1, 29.1]		
Caretaker education (High School or GED)	12.8	[-30.3, 55.9]		
Caretaker education (Some college)	Reference			

B. Chest Feels Tight because of Asthma Symptom

Variables	Fully Adjusted Model Estimate	Fully Adjusted Model 95% CI	Final Model Estimate	Final Model 95% CI
Personal $PM_{2.5}$	Model did not converge		-0.008	[-0.036, 0.019]
Relative Humidity			0.042	[-0.062, 0.145]
Hispanic Ethnicity (No)			2.4	[-1.8, 6.6]
Hispanic Ethnicity (Yes)			0.8	[-3.5, 5.0]
Hispanic Ethnicity (missing)			Reference	
Parent Asthma Status (No)				
Parent Asthma Status (Yes)				
Parent Asthma Status (missing)				
Caretaker education (College)				
Caretaker education (Don't know)				
Caretaker education (Graduate School)				
Caretaker education (High School or GED)				
Caretaker education (Some college)				
Ambient O_3	-0.021	[-0.071, 0.028]	-0.022	[-0.068, 0.024]
Relative Humidity	0.032	[-0.070, 0.135]	0.019	[-0.069, 0.108]
Hispanic Ethnicity (No)	2.2	[-3.0, 7.4]	0.9	[-2.1, 3.9]
Hispanic Ethnicity (Yes)	0.7	[-4.6, 6.0]	-0.3	[-3.3, 2.8]
Hispanic Ethnicity (missing)	Reference			
Parent Asthma Status (No)	-0.784	[-5.834, 4.266]		
Parent Asthma Status (Yes)	-1.734	[-6.633, 3.164]		
Parent Asthma Status (missing)	Reference			
Caretaker education (College)	0.033	[-3.975, 4.042]		
Caretaker education (Don't know)	0.766	[-4.860, 6.392]		
Caretaker education (Graduate School)	-0.086	[-3.752, 3.578]		
Caretaker education (High School or GED)	2.088	[-2.883, 7.060]		
Caretaker education (Some college)	Reference			

C. Rescue Inhaler Use

Variables	Fully Adjusted Model Estimate	Fully Adjusted Model 95% CI	Final Model Estimate	Final Model 95% CI
Personal $PM_{2.5}$	0.002	[-0.021, 0.026]	0.005	[-0.012, 0.022]
Relative Humidity	-0.022	[-0.141, 0.095]	0.001	[-0.095, 0.096]
Hispanic Ethnicity (No)	2.2	[-4.7, 9.0]	1.3	[-2.2, 4.7]
Hispanic Ethnicity (Yes)	-0.4	[-8.0, 7.1]	-0.4	[-3.9, 3.1]
Hispanic Ethnicity (missing)	Reference			
Parent Asthma Status (No)	-0.667	[-6.372, 5.039]		
Parent Asthma Status (Yes)	-0.775	[-7.100, 5.551]		
Parent Asthma Status (missing)	Reference			
Caretaker education (College)	2.648	[-3.050, 8.347]		
Caretaker education (Don't know)	2.217	[-5.202, 9.637]		
Caretaker education (Graduate School)	0.984	[-4.592, 6.561]		
Caretaker education (High School or GED)	2.054	[-8.220, 12.328]		
Caretaker education (Some college)	Reference			
Ambient O_3	-0.002	[-0.020, 0.016]	-0.001	[-0.019, 0.017]
Relative Humidity	0.001	[-0.055, 0.056]	0.001	[-0.051, 0.053]
Hispanic Ethnicity (No)	1.7	[-2.1, 5.4]	0.2	[-2.2, 2.6]
Hispanic Ethnicity (Yes)	0.3	[-3.5, 4.1]	-2.0	[-4.4, 0.4]
Hispanic Ethnicity (missing)	Reference			
Parent Asthma Status (No)	-2.735	[-5.948, 0.476]		
Parent Asthma Status (Yes)	-2.142	[-5.717, 1.432]		
Parent Asthma Status (missing)	Reference			
Caretaker education (College)	1.095	[-1.481, 3.671]		
Caretaker education (Don't know)	1.671	[-2.392, 5.732]		
Caretaker education (Graduate School)	1.081	[-1.895, 4.058]		
Caretaker education (High School or GED)	1.308	[-3.996, 6.612]		
Caretaker education (Some college)	Reference			

Supplement Figure S1. Comparison between forced expiratory volume in one second (FEV_1) collected at recruitment with the Asma-1 BT sensor used in the BREATHE Kit and the and clinic spirometer tested FEV_1 on same or closest previous day to recruitment (units of L/s).

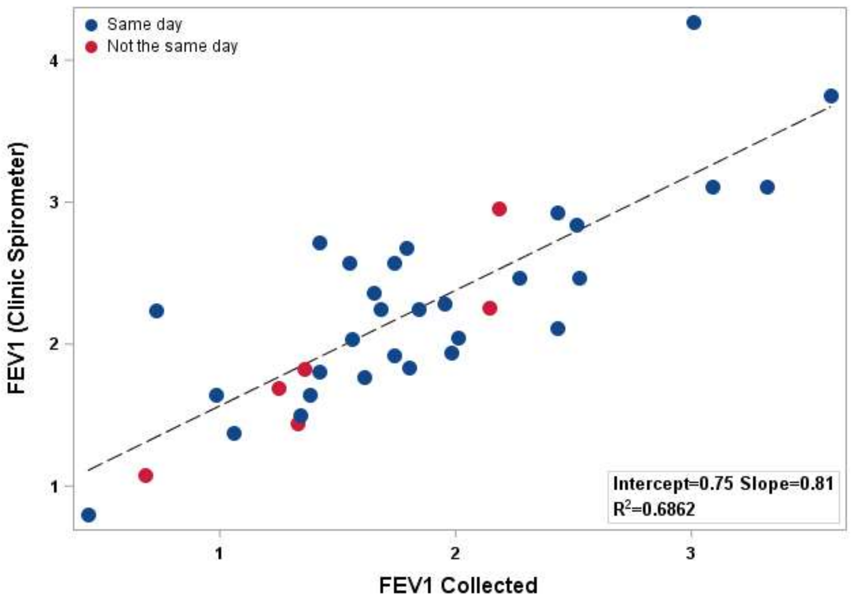

Supplement Figure S2. Rescue inhaler use daily count distribution across all subjects.

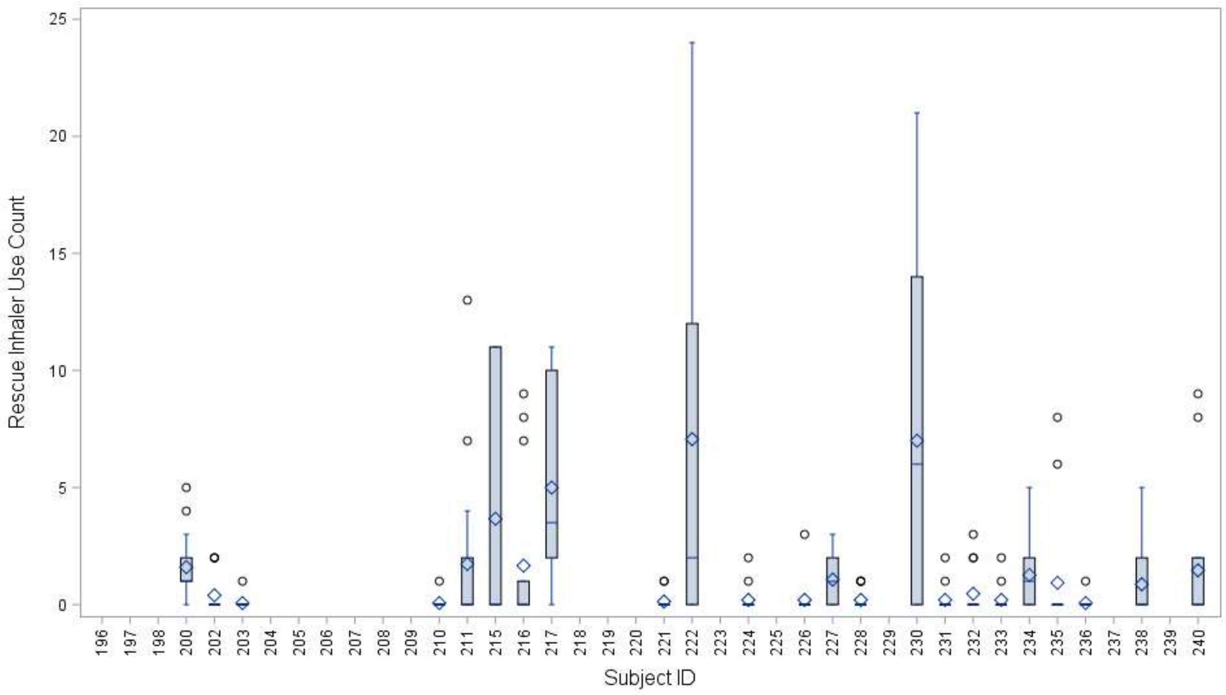

Supplement Figure S3. Distribution of daily average personal (measured), ambient (modeled) and traffic-related (modeled) $PM_{2.5}$ exposures in μg/m³ across all subjects.

Personal

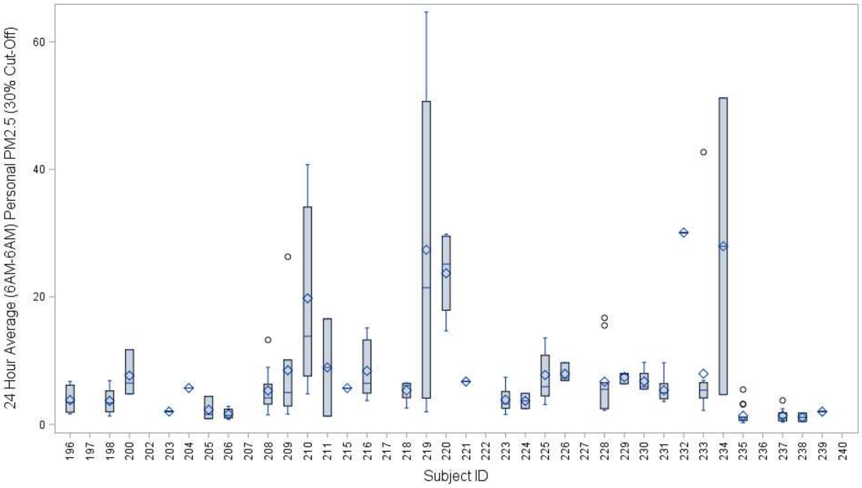

Ambient

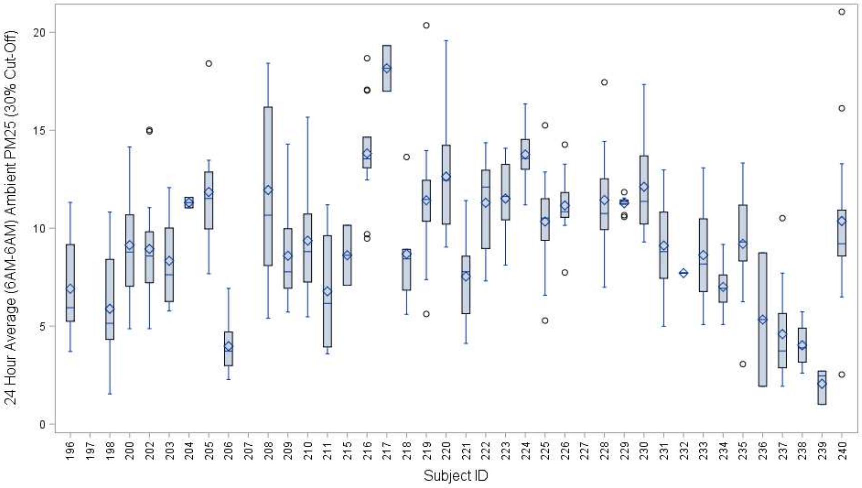

Traffic-related

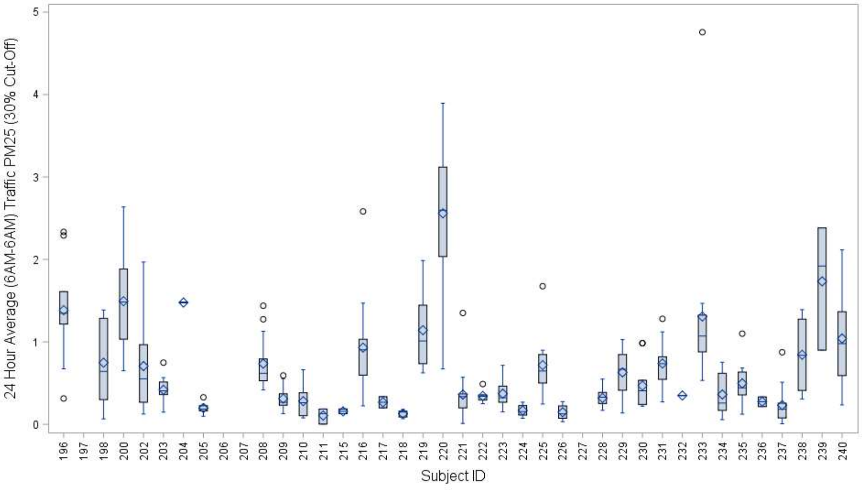

Supplement Figure S4. Results of single- and two-pollutant models (rate ratio and 95% CI per standard deviation increase in exposure) for daily count of rescue inhaler use in relation to traffic-related $PM_{2.5}$, NO, and NO_x exposure in the last 24 hours (lag 0).

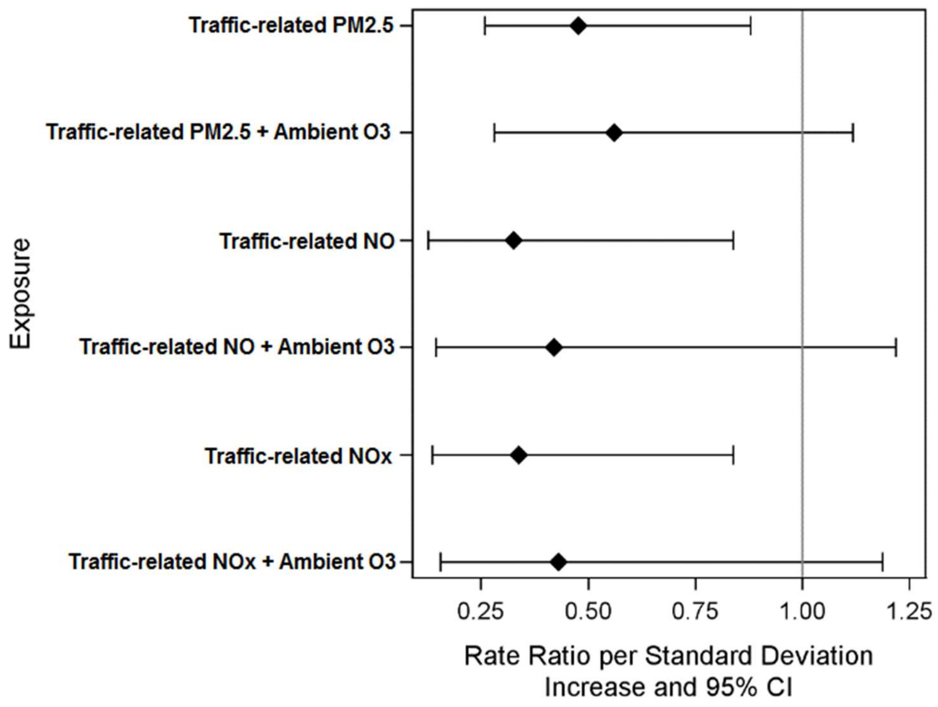

Supplement: Supplementary file 1 [file ijerph-19-03578-s001.zip › ijerph-1585447-supplementary.pdf]
